# Scientific evidence supporting the newly developed one-health labeling tool “Med-Index”: an umbrella systematic review on health benefits of mediterranean diet principles and adherence in a planeterranean perspective

**DOI:** 10.1186/s12967-023-04618-1

**Published:** 2023-10-26

**Authors:** Roberta Zupo, Fabio Castellana, Prisco Piscitelli, Pasquale Crupi, Addolorata Desantis, Enrico Greco, Franca Paola Severino, Manuela Pulimeno, Andrea Guazzini, Tassos C. Kyriakides, Vasilis Vasiliou, Antonia Trichopoulou, Laura Soldati, Carlo La Vecchia, Giovanni De Gaetano, Maria Benedetta Donati, Annamaria Colao, Alessandro Miani, Filomena Corbo, Maria Lisa Clodoveo

**Affiliations:** 1 Department of Interdisciplinary Medicine (DIM), University “Aldo Moro”, Piazza Giulio Cesare 11, 70100 Bari, Italy; 2 Italian Society of Environmental Medicine (SIMA), 20123 Milan, Italy; 3 UNESCO Chair on Health Education and Sustainable Development, Federico II University, 80138 Naples, Italy; 4 Department of Education, Literatures, Intercultural Studies, Languages and Psychology, University of Florence, 50121 Florence, Italy; 5 Yale School of Public Health, New Haven, CT 06510 USA; 6 Academy of Athens, Athens, Greece; 7 Department of Health Sciences, University of Milan, 20122 Milan, Italy; 8 Department of Clinical Sciences and Community Health, Università Degli Studi di Milano, Milan, Italy; 9 Department of Epidemiology and Prevention, IRCCS NEUROMED, 86077 Pozzilli, Italy; 10 Department of Pharmacy-Drug Sciences, University of Bari “Aldo Moro”, 70125 Bari, Italy

**Keywords:** Front of pack (FOP) labeling, Food policy, Med-Index, Mediterranean diet, Planeterranean, Health, One-health

## Abstract

**Background:**

Med-Index is a one-health front-of-pack (FOP) label, based on Mediterranean diet (MedDiet) principles, developed to summarize information about the nutritional properties and related-health benefits of any food as well as its sustainable production processes, and the associated food company’s social responsibility parameters in a new “Planeterranean” perspective. Thus, Med-Index can be adopted in and by any European region and authority as well as worldwide; this is achieved by consumption and cooking of locally available and sourced foods that respect MedDiet principles, both in terms of healthy nutrition and sustainable production. The huge body of scientific evidence about the health benefits of the MedDiet model and principles requires a comprehensive framework to encompass the scientific reliability and robustness of this tool. A systematic review was carried out to examine the association between human health and adherence to MedDiet patterns upon which the “Med-Index” tool was subsequently developed.

**Methods:**

MEDLINE and PubMed databases were searched for eligible publications from 1990 to April 2023. Systematic literature reviews, with or without meta-analysis, of clinical trials and observational studies were screened by two independent investigators for eligibility, data extraction, and quality assessment. English language and the time interval 1990–2023 were applied. A registry code CRD42023464807 was generated on PROSPERO and approved for this search protocol. The corrected covered area (CCA), calculated to quantify the degree of overlap between reviews, gave a slight overlap (CCA = 4%).

**Results:**

A total of 84 systematic reviews out of 6681 screened records were selected. Eligible reviews included studies with predominantly observational designs (61/84, 72.6%%), of which 26/61 referenced studies of mixed observational and RCT designs, while 23/84 (27.4%) were RCT-only systematic reviews. Seventy-nine different entries were identified for health outcomes, clustered into 10 macro-categories, each reporting a statistically significant association with exposure to the MedDiet. Adherence to MedDiet was found to strongly benefit age-related chronic diseases (21.5%), neurological disorders (19%), and obesity-related metabolic features (12.65), followed by CVDs (11.4%), cancer (10.1%), diabetes (7.5%), liver health (6.3%), inflammation (5%), mortality (5%), and renal health (1.2%). The quality of the studies was moderate to high.

**Conclusion:**

In the context of a “Planeterranean” framework and perspective that can be adopted in any European region and worldwide, MedDiet represents a healthy and sustainable lifestyle model, able to prevent several diseases and reduce premature mortality. In addition, the availability of a FOP, such as Med-Index, might foster more conscious food choices among consumers, paying attention both to human and planetary health.

**Supplementary Information:**

The online version contains supplementary material available at 10.1186/s12967-023-04618-1.

## Introduction

The EU Green Deal of late 2019 brought forward the need for action to be proposed as part of the Farm-to-Fork strategy to help consumers make healthy and sustainable food choices [1]. In a follow-up report to the European Parliament and the Council, adopted in May 2020, the European Commission debated the use of additional expressions and presentations to pair with the nutritional declaration. After examining the current, inconsistent state of use of front-of-pack (FOP) labeling among European countries, the Commission assumed its intention of harmonizing mandatory nutrition labeling to provide consumers with more accurate information about the nutritional value of food products. On these lines, a FOP label embodies the right tool for helping consumers appraise a product's nutritional facts immediately, thereby driving their food choices. Further, it stands out as a strategic policy tool in the pursuit of nutrition-related noncommunicable disease prevention, which is key to containing the growing public health burden and healthcare costs as the global population ages.

From a legislative standpoint, pursuant to Article 35 of EU Regulation No. 1168/2011, food business operators are encouraged to add additional forms of expression and/or presentation to the nutrition declaration in graphic forms or symbols, and member states may recommend their use if the conditions set out in the regulation are met. The latter requires the FOP to be supported by a broad scientific basis but also to be well understood and not misleading to the consumer. Also, it must be an objective, non-discriminatory means so as not to create obstacles to goods flow. As such, use by Member States will be fruitful if the average consumer fully understands the beneficial effects based on the wording of the claim [2]. Public institutions, health sector Non Governmental Organisations (NGOs), and/or the private sector have so far developed various FOP systems. The latter include *nutrient-specific* labels [2] providing information on the content of a specific nutrient or *summary* labels [3, 4] providing an assessment of the overall nutritional value. Overall, FOP nutrition labeling schemes vary in presentation (e.g., shape, color, and size), type of public health nutrition message (descriptive, prescriptive, or both), and nutrient focus (e.g., focus on “critical nutrients” or inclusion of positive and negative nutrients). To date, the most common *nutrient-specific* FOP nutrition labeling schemes refer to sodium, total fat, saturated and trans-fat, and total sugars [5]. Yet, although those tools so far proposed or adopted are somewhat informative, the FOP food labeling system lacks a unified tool to be embraced by all Member States, grounded in health principles shared by the entire scientific community against solid, well-acknowledged evidence, while simultaneously fostering an ecological transition strongly advocated by the EU Commission. In other words, a tool that works well to achieve a fair, healthy, and environment-friendly food system that can be readily understood by a consumer of any socioeconomic bracket. To overcome the shortcomings of an inconsistent FOP food system that actually fails to fully address the goals of the Green Deal, particularly Farm to Fork and biodiversity strategies, the Med-Index has been proposed as an innovative, one-health FOP food labeling system that readily informs consumers by iconographic color-supported input on nutritional, environmental, and social sustainability [6, 7]. The adoption of this tool based on the One-health approach—even as an integrative label in addition to others—would add information about healthy properties, environmental sustainability, and social responsibility of any food product, referring to Mediterranean diet principles, in a “Planeterranean” [8] framework and perspective (produce locally and consume locally), thus encouraging producers to produce healthier and more sustainable food [9]. The “Planeterranean” perspective is a newly adopted concept in the scientific community, intended to identify a healthy food model based on foods available in different areas of the world with the nutritional properties of the MedDiet [8].

The rationale of educating consumers on Mediterranean principles is to spread and enhance a sustainable eating pattern that ensures food security, promotes healthy lifestyles, and shares good living practices (i.e., seasonality, conviviality, and an active lifestyle) by facilitating the achievement of the Sustainable Development Goals (SDGs) set by both the United Nations 2030 Agenda and the EU Commission Green Deal. Indeed, as concern for sustainability is growing, the Mediterranean diet has been widely identified as a promising model with benefits for both human and environmental health. In this context, a recent report [10] systematically described the indicators so far used to assess the sustainability of the MedDiet, along with the outputs of their application.

Analysis of the environmental, health-nutritional, economic, and sociocultural dimensions falling under the concept of a sustainable dietary pattern concluded that the MedDiet has a lower environmental impact than Western diets with a carbon footprint between 0.9 and 6.88 kg CO2/d per capita, a water footprint between 600 and 5280 m^3^/d per capita, and an ecological footprint between 2.8 and 53.42 m^2^/d per capita. Regarding the nutritional dimension, MedDiet demonstrated high nutritional quality, scoring better in health and nutrient richness than other diets. Moreover, the cost of MedDiet was similar to that of other diets and ranged from 3.33 to 14.42 €/d per capita.

Widely known, the MedDiet model is mainly based on the consumption of plant-based foods, such as fruits and vegetables, and the supplementation with whole grains, beans, nuts, seafood, lean poultry, and unsaturated fats from extra virgin olive oil. Various cultures around the world, such as Asian countries and northern regions, have already adopted this way of eating, using different varieties of produce, seafood, herbs, and spices. Among others, MedDiet has been defined as a family-friendly, economical, planet-friendly, suitable model for vegans or vegetarians, and a gluten-friendly model.

The nutritional value and health benefits of the Mediterranean diet have been documented since the Seven Countries Study, a study of the relationship between diet and heart disease among 13,000 men living in Greece, Italy, Japan, Finland, the former Yugoslavia, the Netherlands, and the US from 1958 to 1999. Since then, countless studies have shown that this dietary pattern reduces the risk of certain chronic health conditions, such as cardiovascular disease (CVD) and type 2 diabetes, while promoting longevity and improving quality of life. The body of scientific evidence surrounding the multiple health benefits of adhering to the principles of the Mediterranean diet model requires a comprehensive perspective to support the well-rounded scientific reliability of the one-health, Mediterranean Med-Index FOP labeling tool. The objective of this research was to conduct an umbrella systematic review to deeply assess and weigh the relationship between adherence to the MedDiet pattern and human health benefits.

## Methods

### Search strategy, selection criteria, and data extraction

A computer search of the literature on Ovid MEDLINE and the PubMed database (supported by Florentine Bibliotheque, a professional service) has been carried out to assess the strength of the association between adherence or exposure to the Mediterranean diet and human health benefits. The present systematic review followed the Preferred Reporting Items for Systematic Reviews and Meta-Analyses (PRISMA) guidelines, adhering to the 27-item PRISMA checklist [11]. An a priori protocol for search strategy and inclusion criteria was established and registered, with no particular changes to the information provided at the time of registration on PROSPERO, an international prospective registry of systematic reviews, meta-analyses, and umbrella reviews (registry code no. CRD42023425221). Two researchers conducted separate searches in the U.S. National Library of Medicine (PubMed) and across the Medical Literature Analysis and Retrieval System Online (MEDLINE) to find level I evidence articles, i.e., systematic reviews with or without meta-analysis, that explored and reported any meaningful association between exposure to the MedDiet, as assessed by means of adherence scales, dietary patterns, or a priori indices, and outcomes of human health state, with no restrictions set on the type of health endpoint. Accordingly, the main objective was to comprehensively assess how well adherence to the Mediterranean diet pattern was corroborated in the literature to provide health benefits to fortify the scientific validity of the one-health FOP labeling tool Med-Index. The inclusion criteria were systematic review articles—with or without meta-analysis—of clinical trials and observational studies, publication time interval 1990–2023, and English language. No restrictions were placed on the age of the population examined, general health status, country, recruitment context (hospital, community or home care) or setting of the studies included in the systematic reviews (clinical trial, observational study).

The query used in PubMed and MEDLINE used keywords such as "Mediterranean," "diet," and "health" combined through the use of Boolean indicators such as AND and OR as follows: (("diet, Mediterranean"[MeSH Terms] OR ("diet"[All Fields] AND "Mediterranean"[All Fields]) OR "Mediterranean diet"[All Fields] OR ("Mediterranean"[All Fields] AND "diet"[All Fields])) AND ("health"[MeSH Terms] OR "health"[All Fields] OR "health s"[All Fields] OR "healthful"[All Fields] OR "healthfulness"[All Fields] OR "healths"[All Fields] OR ("health status"[MeSH Terms] OR ("health"[All Fields] AND "status"[All Fields]))) AND (2000:2023[pdat]). Two researchers (RZ, FC) sought the records, reviewed the titles and abstracts of the articles retrieved separately and in duplicate, checked the full texts, and selected the articles for inclusion in the study, ruling out all original articles in the first screening phase. Technical reports, letters to the editor, and conference proceedings were also excluded. Inter-rater reliability (IRR) was used to estimate inter-coder agreement, and then κ statistics to measure accuracy and precision. Based on PRISMA concepts and quality assessment steps, a k coefficient of at least 0.9 was obtained in all data extraction steps.

The corrected area covered (CCA), a validated method for quantifying the degree of overlap between two or more reviews, was calculated to aid decision making. According to the algorithm, CCA is expressed as a percentage and is calculated as (N−r)/(rc−r), where N is the number of publications included in the evidence synthesis, r is the number of rows and c is the number of columns. Overlap is classified as very high (CCA > 15%), high (CCA 11–15%), moderate (CCA 6–10%) or slight (CCA 0–5%) [12].

### Data synthesis and risk of bias assessment

Two researchers extracted the following information separately and in duplicate in a piloted form: author(s), year of publication, number of studies analyzed, study design (observational, clinical trial), e-databases searched, study outcome(s) investigated, risk of bias assessment tool, main findings, and meta-analysis (presence/absence). All references selected for retrieval from the databases were managed with the MS Excel data collection software platform by an experienced biostatistician. Finally, the data extracted from the selected studies and stored in the database were structured as evidence tables.

The Risk of Bias in Systematic Reviews (ROBIS) tool was used to assess the risk of bias in the included systematic reviews. The ROBIS involves three steps, including assessment of relevance, identification of concerns about the review process, and judgment of risk of bias [13].

The first stage of the ROBIS tool includes an item, which mainly assesses whether the participants, exposures, comparators, and outcomes match the target question. The responses are “yes”, “no”, “partial”, and “uncertain”. Phase two includes four areas: (1) study eligibility criteria; (2) study identification and selection; (3) data collection and study evaluation; and (4) synthesis and results. Phase two reporting questions are answered with “yes”, “probably yes”, “probably no”, “no”, and “no information”. Based on the response to each reporting question, the bias associated with each domain is judged to be “low”, “high”, or “unclear”. The third stage considers whether the systematic review at risk of bias. At this stage, the following questions were considered: (1) did the interpretation of the results address all the concerns identified in domains 1 to 4; (2) was the relevance of the identified studies appropriately considered in the review's research question; and (3) did the reviewers avoid emphasizing the results based on their statistical significance? The answers to these reporting questions are the same as in phase two. Based on the responses to the stage three questions, the overall risk of bias in systematic reviews was rated as “low”, “high”, or “unclear”. Two researchers independently assessed the risk of bias in all included systematic reviews, and disagreements were resolved through discussion. Any discrepancies during the scoring process were resolved through discussion and consensus. Disagreements between the two investigators on the methodological quality of the included studies were addressed by discussion until a third investigator agreed. All data analyses were performed using R, version 2021.09.1. A biostatistician conducted a quantitative summary of the findings and produced a pie chart of outcome(s) distribution found in association with exposure to the Mediterranean diet across the selected studies and sorted into macro-categories to aid the synthesis of understanding.

Along with our quality assessment, the risk of bias reported in each of the included systematic reviews were extracted, as rated according to different scales depending on each study, and reported in a Additional file 1: Table S1 as overall risk of bias and domain-specific risk of bias (if stated in the study). The table has been provided as Additional file 1: Table S1.

## Results

The first literature search yielded 6681 entries. After excluding duplicates, 4964 were screened for the exclusion of original articles, letters, editorials, and conference proceedings, and then 1717 entries were classified as potentially relevant and selected for the title(s) and abstract analysis. Then, 1624 were excluded because they did not meet the characteristics of the approach or the objective of the review. After reviewing the full text of the remaining 93 papers, a total of 84 reports met the research question and were included in the final qualitative and quantitative analysis [14–97]. The calculation of the CCA obtained a mild degree of overlap across selected studies (CCA = 4%).

**Fig. 1:**
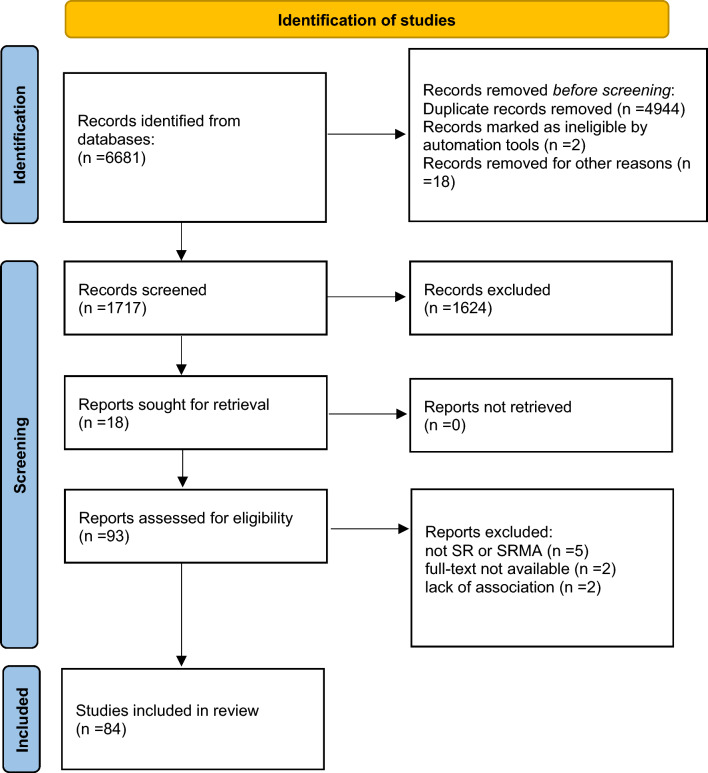
PRISMA flow diagram

**Fig. 2:**
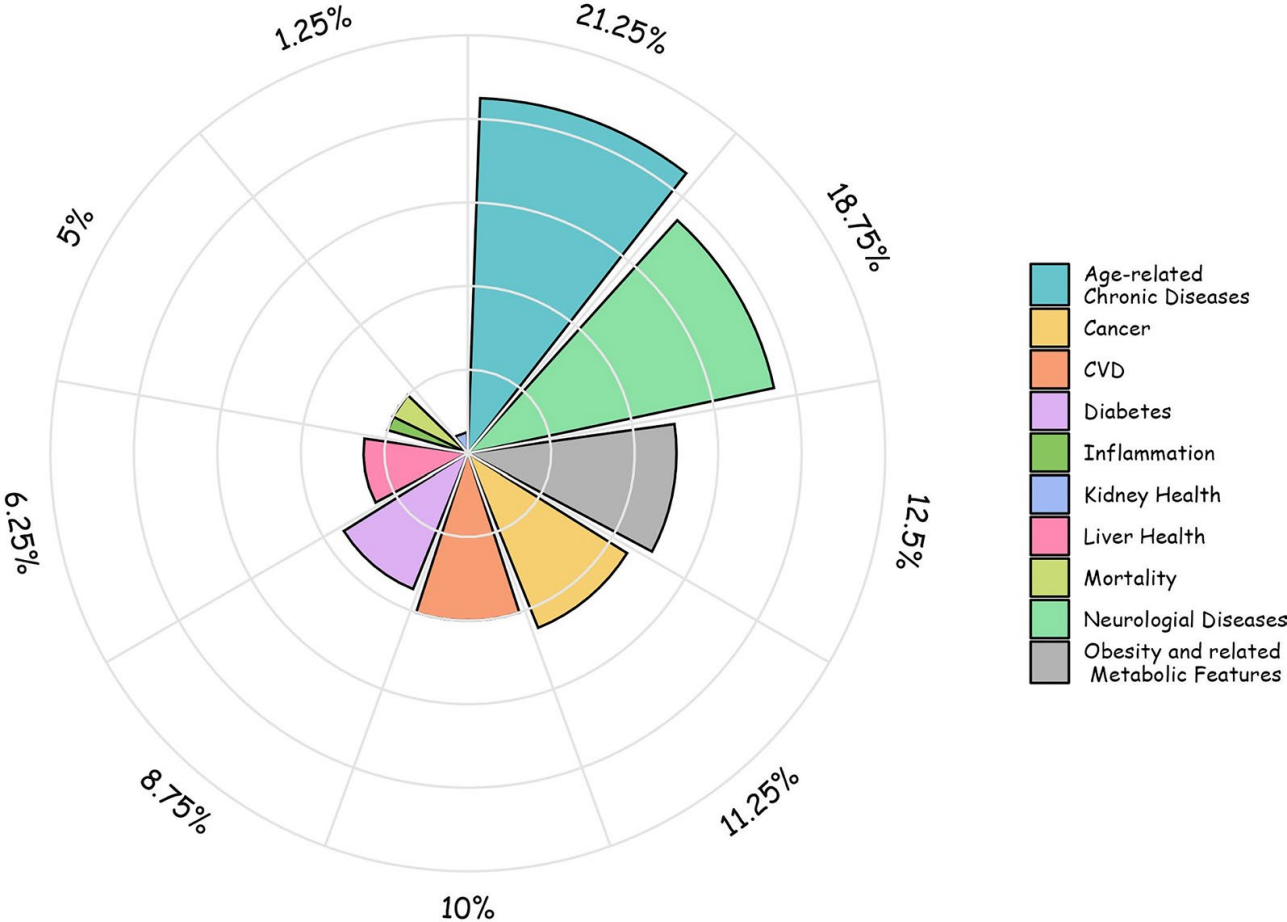
Graphical overview of the results

The flow chart of Preferred Reporting Items for Systematic Reviews and Meta-analyses (PRISMA), showing the number of studies at each stage of paper selection, is shown in Fig. 1. The final study database included 84 systematic review articles, i.e., level I evidence, reporting on the association between adherence to the Mediterranean model and human health outcomes, of which 40 included meta-analyses. Figure 2 shows a graphical overview of the results as a pie chart.

Table 1 gives details of (1) authors; (2) year of publication; (3) design (longitudinal, cross-sectional, RCT); (4) sample size of each report as defined by the number of studies included in each systematic review; (5) outcome(s) examined, and taken individually for the significant association reported in the study; (6) the risk of bias assessment tool(s) used in each study to evaluate the quality assessment; (7) the main findings; and (8) the presence of meta-analysis (yes/no).

**Table 1:** Descriptive of selected observational studies and RCT evaluating the adherence, or the intervention of a Mediterranean diet compared to other diets in relation to various health outcomes

Author details [Refs.]	Year of publication	Number of Studies	Design of studies	Electronic databases searched	Outcome measure(s)	Risk of bias assessment tool	Results	Meta-analysis (yes/no)
Fatima K et al. [14]	2023	15	RCT	MEDLINE Grey literature	Flow-mediated dilation	Cochrane risk of bias tool for RCT	Inverse relationship between endothelial function and intake of MedDiet was observed. Overall, MedDiet increased FMD by 1.39%. There was a significant improvement in endothelial function in both healthy patients and in those with an increased risk of CVD	Yes
Papadopoulou SK et al. [15]	2023	10	Observational	PubMed Cochrane Library Scopus Grey literature	Muscle function	New Castle Ottawa scale (NOS) for cohort studies AXIS tool for cross-sectional studies	MedDiet adherence had, in general, a positive role in muscle mass and muscle function (assessed at 20 m walking test and 15 ft walking test), while the results were less clear about muscle strength	No
Nucci D. et al. [16]	2023	8	Case–control, cohort	MEDLINE Scopus EMBASE	Pancreatic cancer	New Castle Ottawa scale (NOS) for cohort studies	A higher adherence to the MedDiet is associated with a lower risk of pancreatic cancer based on 1,301,320 subjects	Yes
Gregory S et al. [17]	2023	7	Crossectional, cohort	MEDLINE EMBASE CINAHL PsycInfo	White matter hyperintensity volume	NIH Quality Assessment Tool for Observational Cohort and Cross-Sectional Studies	Four studies reported on hippocampal volume, with inconclusive or no associations seen with MedDiet adherence. Two studies found a significant association between higher MedDiet adherence and lower WMHV, while two other studies found no significant associations	No
Townsend RF et al. [18]	2023	93	Cohort, RCT	MEDLINE EMBASE	Dementia Alzheimer's Disease Cognitive decline	Cochrane RoB-2 New Castle Ottawa scale (NOS) for cohort studies	Greater adherence to the MedDiet was positively associated with better global cognitive performance in 5 out of 9 studies (55%). The Mediterranean diet appeared to be more strongly protective for Alzheimer's Disease than for all-cause dementia. A small improvement in cognitive function in response to a Mediterranean style diet was found	No
Moore E et al. [19]	2022	6	RCT	PubMed Cochrane Library MEDLINE	Body Mass Index TNF-α Interleukin-6	NR	The main findings indicate a hypocaloric, fiber dense MedDiet to be a short-term (< 4 months) mitigation strategy to significantly reduce BMI and inflammatory markers amongst overweight/obese adults. Results demonstrate that TNF-α, have been significantly reduced over a 3–4-month MedDiet intervention for overweight/obese adults. Regarding IL-6, short-term (3–4 months) reductions were reported amongst participants who consumed a MedDiet	No
Thackrey E et al. [20]	2022	4	Crossectional, RCT	MEDLINE CENTRAL EMBASE PsycINFO CINAHL ERIC Web of Science	Body Mass Index	NIH Quality Assessment Tool for Before-After (Pre-Post) NIH Quality Assessment Tool for Observational Cohort and Cross-Sectional Studies	Significant weight loss was observed within groups for low-fat diet, MedDiet, fasting, low-calorie diet with fasting, intermittent fasting, or continuous energy restriction, but there were no between-group differences. MedDiet reduced weight at 6 months compared with baseline	No
Sangouni AA et al. [21]	2022	10	RCT	PubMed Web of Science Scopus Google Scholar	AST GGT	Cochrane Risk of Bias Tool for RCT	MedDiet can significantly reduce levels of AST and GGT which are the important markers of liver function. However, MedDiet has no significant effect on ALT	Yes
Pameijer EM et al. [22]	2022	20	RCT	MEDLINE Embase Cochrane Library	Age-related macular degeneration	ROBINS-1 tool AMSTAR-2 tool	A high intake of specific nutrients, the use of antioxidant supplements and adherence to a Mediterranean diet decrease the risk of progression of early to late age-related macular degeneration	No
Angelidi AM et al. [23]	2022	3	RCT	PubMed Embase CINAHL Web of Science	Hepatic fat content Triglycerides	Cochrane Collaboration's risk of bias tool for RCT	A post hoc analysis, including two eligible studies assessing the effect of the Mediterranean, compared to a low-fat diet, irrespective of baseline presence of diabetes, showed strong evidence that the MedDiet significantly reduces hepatic fat content and triglyceride concentrations	No
Luong R et al. [24]	2022	16	RCT, Cohort	Medline Embase DARE Web of Science Scopus	Systolic blood pressure Triglycerides HDL-c	Revised Cochrane risk-of-bias tool for randomized trials (RoB-2) Joanna Briggs Institute (JBI) Critical Appraisal Checklists	The Mediterranean dietary pattern resulted in reduced triglyceride levels and systolic blood pressure and had no effects on diastolic blood pressure and glucose in the short term. MedDiet resulted in increased HDL-cholesterol levels in adults without CVD	No
Sepandi M et al. [25]	2022	10	Crossectional, cohort	Web of knowledge PubMed Cochrane Library Science direct Google Scholar Scopus	Waist circumference Triglycerides HDL-c LDL-c Fasting Blood Glucose HbA1c Total Cholesterol	New Castle Ottawa scale (NOS) for cohort studies	MedDiet score showed an inverse relationship with BMI, WC, TG, total cholesterol, LDL-C, FBG, HbA1c and a direct relationship with HDL-C	No
Noori M et al. [26]	2022	8	Crossectional, cohort	PubMed Scopus Web of Science	Bone mineral density	New Castle Ottawa scale (NOS) for cohort studies	Greater adherence to the MedDiet was associated with a small but important increase in bone mineral density at the lumbar spine, femoral neck, hip, trochanter, and whole body. Adopting a Mediterranean style eating pattern may have modest beneficial effects on bone health	Yes
Gastaldello A et al. [27]	2022	8	Crossectional, cohort	PubMed ScienceDirect Cochrane Library	Age-related macular Degeneration	New Castle Ottawa scale (NOS) for cohort studies	Higher adherence to a Mediterranean eating pattern lowers the odds of developing age-related macular degeneration and decreases the risk of progression to more advanced stages of the disease, establishing the way for preventative measures emphasizing dietary patterns rich in plant-foods	No
Cuevas-Cervera M et al. [28]	2022	16	RCT, observational	PubMed Web of Science ProQuest Scopus Cumulative Index to Nursing & Allied Health Literature (CINAHL) Cambridge Core Oxford Academy	Sleep quality Pain Functional disability Physical health	e-PEDro scale for rating quality of RCT Quality assessment with diverse studies (QuADS)	Levels of pain, functional disability, wellness of sleep, physical and general well-being, and pain location and severity were significantly lower in subjects who followed a MedDiet	No
Gianfredi V et al. [29]	2022	23	Observational	MEDLINE Scopus Web of Science EMBASE Cochrane Library	Pancreatic cancer	AMSTAR-2 tool	Convincing or suggestive evidence was found for a healthy/prudent, plant-based diet, fruit and vegetables, and lower risk of pancreatic cancer	Yes
Lee E et al. [30]	2022	11	Observational	Web of Science MEDLINE CINAHL PsycINFO	Mortality (overall) Breast cancer mortality	New Castle Ottawa scale (NOS) for cohort studies	Among the diet quality indices evaluated, post-diagnostic adherence to MDS, HEI, DASH, and CHFP, and adherence to DASH and CHFP showed significant effects on all-cause mortality and breast cancer mortality, respectively	Yes
Zeraattalab-Motlagh S et al. [31]	2022	14	Cohort	PubMed Scopus Web of Science	Type 2 diabetes	ROBINS-I	Adherence to the MedDiet was inversely related to type 2 diabetes risk in a dose–response manner	Yes
McBean L et al. [32]	2022	10	RCT	Medline EMBASE CENTRAL PsycINFO	Global cognitive function Processing speed	Cochrane Risk of Bias tool	Findings suggest a potential effect of MedDiet on global cognitive function and processing speed	Yes
Bakaloudi DR et al. [33]	2021	58	Observational	PubMed Cochrane CENTRAL Scopus EMBASE Web of Science Google Scholar	Waist circumference HDL-c Triglycerides	New Castle Ottawa scale (NOS) for cohort studies	Waist circumference and triglycerides were significantly lower in the high adherence MedDiet, while HDL cholesterol was significantly higher in the same group. MedDiet may have a positive impact on all parameters of metabolic syndrome	Yes
Tang C et al. [34]	2021	7	Cohort	Embase PubMed Scopus Web of Science Cochrane	Mortality (overall)	New Castle Ottawa scale (NOS) for cohort studies	This meta-analysis of prospective cohort studies provided evidence that adherence to MedDiet improved survival in people with a history of CVD	Yes
Ubago-Guisado E et al. [35]	2021	110	Cohort	MEDLINE Scopus Web of Science	Colorectal cancer Breast cancer	Joanna Briggs Institute Critical Appraisal Tool for Systematic Reviews	Adherence to the MedDiet emerged as a protective factor for colorectal and breast cancer	No
Coelho-Júnior HJ et al. [36]	2021	53	Crossectional, cohort	MEDLINE SCOPUS CINAHL AgeLine	Physical health Global cognitive function	New Castle Ottawa scale (NOS) for cohort studies	Findings of the present study indicated that high adherence to MedDiet was cross-sectionally associated with physical performance and cognitive function. Results of the pooled analysis of longitudinal studies revealed that high adherence to MedDiet reduced the risk of global cognitive decline in non-demented older adults. However, no significant associations between MedDiet adherence and the incidence of mobility problems, mild cognitive impairment, and dementia were found	Yes
Quintela BC et al. [37]	2021	24	Observational	PubMed Embase	eGFR Proteinuria	New Castle Ottawa scale (NOS) for cohort studies	The consumption of fruit, vegetables and dietary fiber (DASH and MedDiet) revealed low risk associations for chronic kidney disease, being recommended models to reduce the occurrence and progression of the disease	No
Silveira EA et al. [38]	2021	7	RCT, cohort	PubMed Scopus Scielo Web of Science	Body Mass Index Body fat Blood pressure Fasting Blood Glucose C-Reactive Protein Total Cholesterol	NR	The MedDiet had beneficial changes in weight loss and maintenance, reduction of body fat and inflammatory factors. MedDiet had greater long-term favorable effects on CVD risk factors like BMI, blood pressure, fasting blood glucose, total cholesterol and inflammatory markers such as CRP in individuals with obesity	No
Gibbs J et al. [39]	2021	41	RCT	MEDLINE Embase Web of Science	Systolic blood pressure Diastolic blood pressure	RoB-2: a revised tool for assessing risk of bias in randomized trials	Consumption of the Mediterranean diet was associated with a mean reduction in SBP and DBP compared with the consumption of comparator diets	Yes
Kadam I et al. [40]	2021	20	Observational	PubMed CINAHL Scopus	Body Mass Index Waist circumference	NIH Quality Assessment Tool for Observational Cohort and Cross-Sectional Studies	Adherence to a MedDiet was associated with a lower risk of excessive WC and lower risk of obesity	No
Hart MJ et al. [41]	2021	69	Observational	Embase CINAHL Global Health MEDLINE	C-Reactive Protein Interleukin-6	NIH Quality Assessment Tool for Observational Cohort and Cross-Sectional Studies	Adherence to healthy, Mediterranean and anti-inflammatory dietary scores, appear to be associated with lower inflammatory status cross sectionally. The most frequently assessed biomarkers were CRP and/or IL-6	No
Bianchi VE et al. [42]	2021	79	RCT	MEDLINE EMBASE Cochrane Library	Cognitive decline Alzheimer’s disease Parkinson disease	NR	MedDiet, nutritional support, and calorie-controlled diets play a protective effect against cognitive decline, Alzheimer’s disease (AD), Parkinson disease (PD) while malnutrition and insulin resistance represent significant risk factors	No
Schönenberger KA et al. [43]	2021	12	RCT, observational	MEDLINE Embase CINAHL	Rheumatoid Arthritis	RoB 2 ROBINS-I	Vegetarian, vegan, and MedDiet might be beneficial for some RA patients. However, due to lack of blinding, effects on the patient-reported outcome pain might be biased	Yes
Klonizakis M et al. [44]	2021	20	RCT	PubMed Embase Cochrane Library	Body Mass Index Waist circumference Body fat ALT Visceral adipose tissue	RoB 2	When the comparative effectiveness of an isocaloric MedDiet was compared against the DASH diet for 12 weeks, all of the anthropometric indices (body weight, waist circumference, body fat, and visceral adipose tissue, and additionally ALT were improved in the MedDiet arm	Yes
George ES et al. [45]	2021	30	Observational	MEDLINE CINAHL Embase	Hepatocellular Cancer	The Academy of Nutrition and Dietetics Evidence Analysis Library (EAL) Quality Criteria Checklist	Higher adherence to the MedDiet pattern, Alternative Healthy Eating Index-2010, the Urban Prudent Dietary Pattern, the Traditional Cantonese Dietary Pattern, intake of vegetables, wholegrains, fish, poultry, coffee, macronutrients such as monounsaturated fats and micronutrients such as vitamin E, vitamin B9, β-carotene, manganese and potassium were associated with a reduced risk of hepatocellular cancer	No
Shannon OM et al. [46]	2020	14	RCT	Medline Embase Scopus	Flow-mediated dilation	Cochrane risk of bias tool to assess the risk of bias	MedDiet interventions improve endothelial function in adults, suggesting that the protective effects of the MedDiet are evident at early stages of the atherosclerotic process with important implications for the early prevention of CVD	Yes
Limongi F et al. [47]	2020	45	RCT, cohort	PubMed Scopus	Cognitive decline Global cognitive function	New Castle Ottawa scale (NOS) for cohort studies	Overall, the studies showed that the MedDiet has some protective effects on cognitive decline. As far as cognition domains were concerned, the MedDiet was associated only with improved global cognition. There was no evidence that it has a beneficial effect on dementia	No
Molina-Montes E et al. [48]	2020	26	Observational, RCT	PubMed	Cancer-related mortality	Egger’s test and visual inspection of the funnel plots	The association between adherence to the MedDiet and cancer mortality reached statistical significance	Yes
Papadaki A et al. [49]	2020	57	RCT	PubMed Embase CINAHL Web of Science	Stroke Body Mass Index LDL-c HDL-c HOMA-IR Hepatic fat mass Interleukin-6 Fasting Blood Glucose Triglycerides C-Reactive Protein Waist circumference Flow-mediated dilation AST Blood pressure	ROBINS-I	The MedDiet resulted in greater beneficial changes in 18 of 28 metabolic syndrome components and risk factors (body weight, body mass index, waist circumference, systolic and diastolic blood pressure, glucose, insulin, homeostatic model assessment of insulin resistance (HOMA-IR) index, total-, low-density lipoprotein (LDL)- and high-density lipoprotein (HDL)-cholesterol, triglycerides, alanine transaminase, hepatic fat mass, C-reactive protein, interleukin-6, tumor necrosis factor-a, and flow-mediated dilatation) and lower risk of cardiovascular disease incidence and stroke. Findings support MedDiet’s beneficial effect on all components and most risk factors of the metabolic syndrome, in addition to cardiovascular disease and stroke incidence	Yes
Granic A et al. [50]	2020	28	Observational, RCT	MEDLINE Embase Web of Science Cochrane Library	Muscle function Muscle strength Muscle mass Sarcopenia	RoB 2	Higher intake of fatty fish was beneficial for muscle strength (GS) in both men and women, whilst total fish (white/shell/fatty) and vegetables intake were beneficial for muscle function (gait speed and chair rises, respectively) only in women. Consuming recommended levels of vegetables, a day (≥ 5 servings/day) was associated with higher muscle mass, and more frequent nuts consumption per week reduced the odds of sarcopenia by 30% only in women. In longitudinal associations with all participants, daily intake of soy products, green or yellow vegetables was associated with lower decline in muscle strength (knee extension strength), whilst intake of ≥ 3 servings/day (≥ 85.1 g/day) of poultry or fish was associated with 0.8%–1.2% higher muscle mass	No
Moazzen S et al. [51]	2020	21	Observational	PubMed EMBASE Web of Science Cochrane Library	Gastrointestinal Cancer	New Castle Ottawa scale (NOS)	The highest-quality diets were significantly associated with reduced risk of upper gastrointestinal cancers, achieving odds ratios of 0.59 for the Diet Inflammatory Index, pooling the findings from nine studies, and 0.72 for the MedDiet score	Yes
Abbate M et al. [52]	2020	21	RCT	PubMed	Body Mass Index Triglycerides C-Reactive Protein Waist circumference HOMA-IR HDL-c Total cholesterol LDL-c Blood pressure waist-to-hip ratio Fating Blood Glucose HbA1c Insulin	American Dietetic Association Quality Criteria Checklist	A MedDiet intervention without physical activity, decreased both systolic and diastolic blood pressure, major CV events rate and risk of developing type 2 diabetes	No
Dianatinasab M et al. [53]	2020	10	Observational	PubMed Scopus Web of Science	Breast Cancer	New Castle Ottawa scale (NOS) for cohort studies	This meta-analysis provides supporting evidence for the association between MedDiet decreased risk of invasive ductal carcinoma and invasive ductal carcinoma of the breast	Yes
Rees K et al. [54]	2020	30	RCT	Cochrane Library MEDLINE EMBASE Web of Science	Stroke CV mortality Mortality (overall) Triglycerides LDL-c Diastolic Blood Pressure Systolic Blood Pressure Total cholesterol	Cochrane Handbook for Systematic Reviews of Interventions	Clinical endpoints were reported in two trials where there was moderate quality evidence for a reduction in strokes for primary prevention, and low-quality evidence for a reduction in total and CVD mortality in secondary prevention. There was low-quality evidence for a possible small reduction in total cholesterol and moderate-quality evidence for a reduction in systolic and diastolic blood pressure. A possible small reduction in LDL cholesterol and triglycerides	No
Ge L et al. [55]	2020	121	RCT	Medline Embase CINAHL AMED CENTRAL	Body Mass Index DBP SBP	GRADE (grading of recommendations, assessment, development, and evaluation) approach	Compared with usual diet, moderate certainty evidence supports modest weight loss and substantial reductions in systolic and diastolic blood pressure for low carbohydrate (e.g., Atkins, Zone), low fat (e.g., Ornish), and moderate macronutrient (e.g., DASH, Mediterranean) diets at six but not 12 months	Yes
Parvizian MK et al. [56]	2020	11	Observational	MEDLINE Embase Web of Science CINAHL LILACS AMED Cochrane Library	Chronic obstructive pulmonary disease	National Institutes of Health Quality Assessment Tools (NIH-QATs)	Consumption of a healthy dietary pattern such as the MedDiet was associated with a lower risk of COPD	Yes
Genel F et al. [57]	2020	5	Observational, RCT	MEDLINE EMBASE Cochrane Library	Body Mass Index Interleukin-6 C-Reactive Protein	RoB-2 checklist per RCT Cochrane Handbook’s ROBINS-I tool for cohort	Low-level evidence suggests that low-inflammatory diets or supplements compared to usual diets are associated with greater weight loss and improvement in inflammatory biomarkers	Yes
Altun A et al. [58]	1 Sep 2019	26	Observational, RCT	Medline Embase PsychInfo Scopus Google Scholar	Depression	NIH quality assessment tool w	The majority (85%) of observational studies support the evidence that the Mediterranean dietary pattern is associated with reductions in depressive incidence and all intervention studies echoed these findings	No
Lassale C et al. [59]	2019	41	Observational	Medline Embase PsychInfo	Depression	New Castle Ottawa scale (NOS)	The most compelling evidence was found for the MedDiet and incident depression, with a combined relative risk estimate of highest vs. lowest adherence category. Adhering to a healthy diet, in particular a traditional MedDiet, or avoiding a pro-inflammatory diet appears to confer some protection against depression in observational studies	Yes
Chapman NA et al. [60]	2019	18	Observational, RCT	Cochrane Library Embase Google Scholar Medline Scopus	Age-related macular degeneration	Oxford Centre for Evidence-based Medicine 2011 Levels of Evidence	Adherence to a MedDiet had decreased risk of AMD progression	Yes
Samadi M et al. [61]	2019	26	Observational	PubMed Scopus Web of science	Alzheimer’s Disease	New Castle Ottawa scale (NOS) for cohort studies	Our findings showed that adherence to healthy diet can decrease oxidative stress and inflammation and accumulation of amyloid-β and consequently can decrease the risk of Alzheimer’s Disease	No
Saeed N et al. [62]	2019	6	RCT	MEDLINE EMBASE Scopus Google Scholar	Body Mass Index Hepatic fat content	Downs and Black checklist	Reduction in hepatic steatosis (HS) was statistically significant in 3/5 MedDiet, one low-carbohydrate, one intermittent fasting (IF) and 1/2 low fat (LF) diet interventions. A total of 3/5 studies using MedDiet, 1/2 LF interventions, and the one IF intervention demonstrated significant reductions in weight. In conclusion, there appears to be most data in support of MedDiet-based interventions, though further randomized trials are needed to assess comparative effectiveness for NAFLD	No
Reijnders IF et al. [63]	2019	82	Observational, RCT	Medline Embase PubMed Cochrane Library Web of Science Google Scholar	Resistance of the uterine and umbilical arteries	ErasmusAGE tool	Adequate nutrition in the first trimester, periconceptional folic acid supplement intake and strong adherence to a Mediterranean diet, were all associated with a lower resistance of the uterine and umbilical arteries in the second and third trimester	No
Ajjarapu AS et al. [64]	2019	26	Cohort	PubMed	eGFR	NR	Adherence to the Dietary Approaches to Stop Hypertension (DASH) and Mediterranean diets were significantly associated with a decreased risk of CKD in most of the studies	No
Wu XY et al. [65]	2019	17	Observational	MEDLINE EMBASE PSYCINFO	HRQoL	New Castle Ottawa scale (NOS) for cohort studies	A good adherence to Mediterranean dietary pattern among children and adolescents is associated with better HRQoL	Yes
Xiao Y et al. [66]	2019	69	Observational	PubMed Embase Cochrane Library	Breast cancer	New Castle Ottawa scale (NOS) for cohort studies	The prudent dietary pattern, comparing high vs. low groups, was associated with a reduced risk (overall 18% decrease) of breast cancer in both case–control (30% decreased risk) and cohort studies (11% decrease)	Yes
Govindaraju T et al. [67]	2018	15	Observational, RCT	Medline Embase Psychinfo Cochrane Library Cinhal Web of Sciences Scopus	QoL	Cochrane Collaboration risk of bias tool	Healthy dietary patterns were associated with better self-rated health and QoL in one or more domains, and adherence to healthy dietary patterns like the MedDiet were significantly associated with improvement in at least one of the QoL domains	Yes
Wang Y et al. [68]	2018	6	Observational	MEDLINE EMBASE Cochrane Central Register of Controlled Trials	Frailty	New Castle Ottawa scale (NOS) for cohort studies	A higher adherence to MedDiet is associated with a lower risk of frailty in old people	Yes
Forsyth C et al. [69]	2018	4	Cohort, RCT	Medline Scopus CINAHL Cochrane Library	Rheumatoid arthritis	Cochrane Collaboration’s tool NIH quality assessment tool	This review has identified beneficial effects of the MedDiet in reducing pain and increasing physical function in people living with rheumatoid arthritis	No
Radd-Vagenas S et al. [70]	1 Mar 2018	9	RCT	MEDLINE CINAHL EMBASE PubMed PsycINFO Web of Science	Brain-derived neurotrophic factor Global cognitive function Working memory Verbal and visual memory Language Executive function	PEDro scale for rating quality of randomized controlled trials	The risk of low-plasma brain-derived neurotrophic factor was reduced by 78% (OR = 0.22; 95% CI: 0.05, 0.90) in those who consumed a Mediterranean diet compared to control diet at 3 y in this trial. There were significant ESs, ranging from 0.32 to 1.66, in favor of a MedDiet for 8 test outcomes related to global cognition, working memory, verbal and visual memory, visuospatial, language, and executive function domains	No
Kojima G et al. [71]	2018	4	Cohort	Embase MEDLINE CINAHL PsycINFO Cochrane Library	Frailty	New Castle Ottawa scale (NOS) for cohort studies	Greater adherence to a MedDiet is associated with significantly lower risk of incident frailty in community-dwelling older people	Yes
Morales-Ivorra I et al. [72]	2018	3	RCT, crossectional	EMBASE	Osteoarthritis	New Castle Ottawa scale (NOS) for cohort studies	Positive association between a higher adherence to a MedDiet and the quality of life of participants suffering OA. Three studies included in this systematic review demonstrated some relation between osteoarthritis and a Mediterranean diet	No
Nomikos T et al. [73]	2018	14	Crossectional, RCT	PubMed	Platelet-activating factor	NR	Preliminary results indicate that the characteristic “healthy” components of the MedDiet, especially, cereals, legumes, vegetables, fish and wine can favorably modulate the pro-inflammatory actions of PAF and regulate its metabolism	No
Mayr HL et al. [74]	2018	11	Observational, RCT	PubMed Scopus Web of Science Cochrane Library	TNF-α C-Reactive Protein Interleukin-6	Academy of Nutrition and Dietetics Quality Criteria Checklist: Primary Research	Two trials conducted in Spain demonstrated significant reductions in C-reactive protein with a MedDiet. Four observational studies reported significant inverse associations between Mediterranean-type diet scores and inflammatory cytokines. Five clinical trials (4 in non-Mediterranean countries) demonstrated nonsignificant reductions, and 2 trials conducted in Spain demonstrated significant reductions in C-reactive protein with a MedDiet. One cross-sectional study highlighted that for each unit of increase in Mediterranean diet score, there was 1.9% reduction in the average plasma IL-6 levels when controlling for potential confounders. One cross-sectional study highlighted that Mediterranean diet score was significantly inversely associated with coronary venous blood TNF-α levels when adjusting for potential confounding	Yes
Padilha GR et al. [75]	2018	12	Observational, RCT	Medline Embase Cochrane Library	TNF-α QoL Systolic function Interleukin-6 Left ventricular ejection fraction Left atrial ejection fraction	Cochrane Collaboration Risk of Bias Tool	The MedDiet demonstrated positive correlation with factors of secondary prevention of HF but need more RCT and cohort studies to confirm this effect. The Mediterranean diet had a correlation with inflammation (IL-6 and TNF-α), quality of life and cardiac function but just on cross-sectional studies. A positive association was found between higher adherence scores to a Mediterranean diet and systolic function, left ventricular ejection fraction and left atrial ejection fraction	No
Wong MY et al. [76]	2018	31	Observational, RCT	PubMed Embase Medline Cochrane Library	Diabetic retinopathy	Cochrane Collaboration Risk of Bias Tool	Dietary fiber, oily fish, a Mediterranean diet and a reduced caloric intake are associated with lower risk of Diabetic Retinopathy	No
Malakou E et al. [77]	2018	11	RCT	Medline Embase CINAHL Web of Science	Body Mass Index Waist circumference Systolic Blood Pressure Diastolic Blood Pressure HOMA-IR Triglycerides Total cholesterol HDL-cholesterol Fasting Blood Glucose	Cochrane Collaboration Risk of Bias Tool	There was strong evidence of a beneficial effect of promoting the MedDiet and physical activity (PA) on body weight, body mass index, waist circumference, systolic and diastolic blood pressure, HOMA-IR index, blood glucose, triglycerides, total cholesterol (− 6.30 mg/dL, 95% CI − 9.59, − 3.02) and HDL-cholesterol. There was no evidence of an effect on insulin concentrations. The data presented here provide systematically identified evidence that concurrently promoting the MedDiet and PA is likely to provide an opportunity for metabolic risk reduction	Yes
Mijatovic-Vukas J et al. [78]	2018	40	RCT	PubMed Medline CINAHL Science Direct EMBASE	Gestational Diabetes Mellitus	Quality Criteria Checklist from American Dietetic Association	Diets resembling MedDiet/DASH diet as well as higher PA levels before or in early pregnancy were associated with lower risks or odds of gestational Diabetes Mellitus	Yes
Aridi Y.S. et al. [79]	2017	31	Observational, RCT	MEDLINE Sciencedirect Scopus CINAHL	Dementia Global cognitive function Alzheimer’s disease	Quality Criteria Checklist from American Dietetic Association	Cohort studies in the Mediterranean region and randomized controlled trials showed more cohesive outcomes of the beneficial effect of the MedDiet on cognitive function. Although more standardized and in-depth studies are needed to strengthen the existing body of evidence, results from this review indicate that the Mediterranean diet could play a major role in cognitive health and risk of Alzheimer’s disease and dementia	No
Anton SD et al. [80]	2017	16	RCT	PubMed Cochrane Library Web of Science	Body Mass Index	Cochrane risk of bias scale	After 12 months, the traditional MedDiet produced an average weight loss of 8.7% (7.4 kg) and the low-carbohydrate Mediterranean diet produced an average weight loss of 10.3% (8.9 kg)	No
Bloomfield HE et al. [81]	2016	56	RCT	MEDLINE CINAHL Cochrane Library	Myocardial infarction Type 2 diabetes Breast cancer CV mortality	AHRQ tool	Evidence suggests that a MedDiet with no restriction on fat intake may reduce the incidence of cardiovascular events, breast cancer, and type 2 diabetes mellitus but may not affect all-cause mortality. A MedDiet reduced the risk for a new myocardial infarction and cardiovascular death	Yes
Liyanage T et al. [82]	2016	6	Observational	MEDLINE EMBASE Cochrane Library	Stroke Heart failure	Cochrane Collaboration Risk of Bias Tool	The MedDiet may protect against vascular disease. However, both the quantity and quality of the available evidence is limited and highly variable. When data for all studies were combined there was evidence of protection against major vascular events (RR 0.63, 95% confidence interval 0.53–0.75), coronary events (0.65, 0.50–0.85), stroke (0.65, 0.48–0.88) and heart failure (0.30, 0.17–0.56) but not for all-cause mortality (1.00, 0.86–1.15) or cardiovascular mortality (0.90, 0.72–1.11)	Yes
Petersson SD et al. [83]	2016	25	Observational, RCT	PubMed Embase CINAHL CENTRAL PsycINFO	Global cognitive function	NR	Adherence to the MedDiet is associated with better cognitive performance	No
Neale EP et al. [84]	2016	17	RCT	Scopus PubMed Web of Science Cochrane Library	C-Reactive Protein	GRADE	Consumption of a healthy dietary pattern was associated with significant reductions in CRP. Non-significant changes were found for all other biomarkers	Yes
Garcia M et al. [85]	2016	29	RCT	PubMed EMBASE Web of Science CINAHL	Waist circumference Triglycerides Fasting Blood Glucose Systolic Blood Pressure Diastolic Blood Pressure	Cochrane Collaboration Risk of Bias Tool	There were significant effects in favor of the MedDiet for waist circumference, triglycerides, blood glucose, systolic blood pressure, and diastolic blood pressure. The Med diet was significantly beneficial when the intervention was longer in duration, was conducted in Europe, used a behavioral technique, and was conducted using small groups The traditional Med diet had significant beneficial effects on five of the six metabolic risk factors	Yes
Potter J et al. [86]	2016	64	Cohort	Scopus MEDLINE Medline in Process EMBASE CINAHL	Breast cancer Colorectal cancer Head and neck cancer	New Castle Ottawa scale (NOS) for cohort studies	This body of evidence suggests that higher diet quality, as measured by several indices, is associated with reduced risk of postmenopausal breast cancer, CRC, and head and neck cancer. All-sites cancer risk and cancer mortality were not consistently associated with any of the diet quality scores using any of the indices. All-sites cancer risk and cancer mortality were not consistently associated with any of the diet quality scores using any of the indices	No
Steck SE et al. [87]	2015	12	Observational	MEDLINE	Colorectal cancer	NR	Comparing highest to lowest score groups, higher MDSs were associated with an 8–54% lower CRC risk, and higher HEI scores were associated with a 20–56% lower CRC risk. More proinflammatory diet scores were associated with a 12–65% higher CRC risk compared with more anti-inflammatory diets in studies that used the DII	No
Aljadani H et al. [88]	2015	16	Cohort	MEDLINE EMBASE CINAHL Scopus	Body Mass Index	JBI-MAStARI tool	Higher diet quality is associated with relatively lower prospective weight gain, as well as a lower risk of becoming overweight or obese	No
Koloverou E et al. [89]	2014	10	Cohort	PubMed Embase Cochrane Library	Type 2 diabetes	NR	Higher adherence to the MedDiet was associated with 23% reduced risk of developing type 2 diabetes, combined relative risk for upper versus lowest available centile	Yes
Grosso G et al. [90]	2014	58	Observational	PubMed	Body Mass Index Type 2 diabetes C-Reactive Protein Interleukin-6 Fasting Blood Glucose HOMA-IR HDL-c Metabolic syndrome Waist-to-hip Ratio Flow-mediated dilation	NR	The results here reviewed support the healthy role of MedDiet in both primary and secondary prevention of CVD diseases. A protective role of the MedDiet toward several chronic diseases such as Metabolic Syndrome, diabetes, obesity, CVD events, as well as improvement of lipid profile, hypertension in prehypertensive individuals, coagulation markers, inflammatory markers in subjects with abdominal obesity	No
Kontogianni MD et al. [91]	2014	7	Observational, RCT	PubMed Embase Scopus	Stroke	NR	Consistent, protective effect of higher adherence to the MedDiet on stroke incidence	Yes
Singh B et al. [92]	2014	5	Observational	MEDLINE EMBASE PsycInfo	Mild Cognitive Impairment Alzheimer’s disease	New Castle Ottawa scale (NOS) for cohort studies	Higher adherence to the MedDiet was associated with reduced risk of MCI and Alzheimer’s disease. The subjects in the highest MedDiet tertile had 33% less risk (adjusted HR = 0.67; 95% CI 0.55–0.81; P < 0.0001) of cognitive impairment (MCI or Alzheimer’s disease) as compared to the lowest MedDiet score tertile. Among cognitively normal individuals, higher adherence to the MedDiet was associated with a reduced risk of MCI and Alzheimer’s disease	Yes
Sofi F et al. [93]	2010	18	Cohort	MEDLINE EMBASE Web of Science Cochrane Library	Mortality (overall) CV mortality Alzheimer’s disease CHD Stroke	NR	A 2-point increase in adherence to the Mediterranean diet was associated with a significant reduction of overall mortality [relative risk (RR) = 0.92; 95% CI: 0.90, 0.94], cardiovascular incidence or mortality, cancer incidence or mortality, and neurodegenerative disease	Yes
Verberne L et al. [94]	2010	12	Observational	Scopus PubMed	Breast cancer Oral cancer Esophageal cancer Laryngeal cancer	NR	The existing evidence from observational studies collectively suggests that there is a “probable” protective role of the Mediterranean diet toward cancer in general. Specific results for several outcomes such as different cancer sites deserve additional evidence. This favorable effect of the MedDiet on cancer reduction is of public health relevance, given the tendency of modern societies to shift toward a more U.S. and Northern European dietary pattern	No
Brown T et al. [95]	2009	39	RCT	MEDLINE	Triglycerides HDL-c DBP Fasting Blood Glucose	NR	A MedDiet with behavior therapy vs. a standard low-fat diet was associated with significant weight change at 24 months. There were significant improvements in total cholesterol and HDL cholesterol, triglycerides, glucose and diastolic blood pressure at 24 months	No
Mente A et al. [96]	2009	189	Cohort, RCT	MEDLINE	CHD	NR	Strong evidence supports valid associations of protective factors, including intake of vegetables, nuts, and “Mediterranean” and high-quality dietary patterns with CHD	No
Buckland G et al. [97]	2008	21	Observational, RCT	MEDLINE	Body Mass Index	NR	13 studies reported that MedDiet adherence was significantly related to less overweight/obesity or more weight loss. Eight studies found no evidence of this association	No

N = 84

All eligible reviews were published in English and involved studies with predominantly observational settings (61/84, 72.6%%), of which 26/61 referred to studies with mixed observational and RCT settings, while 23/84 (27.4%) were systematic reviews including only RCT-type clinical trials. Seventy-nine different items were identified for health outcomes and grouped into 10 macro-categories, each of which reported a meaningful significance of the association with MedDiet exposure or adherence. The grouping and specifics of each health outcome category were featured as follows: (i) age-related chronic diseases (age-related macular degeneration, bone mineral density, chronic obstructive pulmonary disease, diabetic retinopathy, frailty, health-related quality of life, muscle function, osteoarthritis, physical health, quality of life, rheumatoid arthritis, sleep quality, muscle strength, pain, muscle mass, functional disability, sarcopenia); (ii) neurological diseases (brain-derived neurotrophic factor (BDNF), cognitive decline, dementia, depression, global cognitive function, Alzheimer’s disease (AD), stroke, white matter hyperintensity, processing speed, working memory, Parkinson's disease (PD), verbal and visual memory, language, executive function); (iii) obesity and related metabolic features (body mass index (BMI), waist circumference (WC), body fat, HDL-cholesterol (HDL-c), LDL-cholesterol (LDL-c), visceral adipose tissue (VAT), total cholesterol (TC), metabolic syndrome, waist–to-hip ratio (WHR), triglycerides); (iv) CVD (left ventricular ejection fraction, left atrial ejection fraction, blood pressure, diastolic blood pressure (DBP), systolic blood pressure (SBP), resistance of the uterine and umbilical arteries, myocardial infarction, flow-mediated dilation (FMD), coronary heart disease (CHD)); (v) cancer (laryngeal cancer, colorectal cancer (CRC), gastrointestinal cancer, breast cancer, pancreatic cancer, oral cancer, head and neck cancer, esophageal cancer); (vi) diabetes (gestational diabetes mellitus, type 2 diabetes mellitus (T2DM), fasting blood glucose (FBG), HOMA-IR, HbA1c, insulin); (vii) inflammation (Platelet-Activating Factor (PAF), TNF-alpha, interleukin-6 (IL-6), C-reactive protein (CRP); (viii) liver health (aspartate aminotransferase (AST), gamma-glutamyl transferase (GGT), alanine transaminase (ALT), hepatic fat content); ix) mortality (breast cancer-mortality, cardiovascular mortality, cancer-related mortality, overall mortality); (x) kidney health (proteinuria, estimated glomerular filtration rate (eGFR).

Twenty-two different databases used through the studies were identified (Medline, EMBASE, Pubmed, Cochrane Library, Scopus, Web of science, CINAHL, Psycinfo, Google Scholar, CENTRAL, Science Direct, Grey Literature, AMED, ERIC, Web of Knowledge, DARE, ProQuest, Cambridge Core, Oxford Academy, AgeLine, Scielo, and LILACS), distributed as follows: Medline (N = 51 entries, 17.17%), EMBASE (N = 51 entries, 17.17%), Pubmed (N = 38, 12.79%), Cochrane Library (N = 31 entries, 10.43%), Scopus (N = 31 entries, 10.43%), Web of science (N = 30, 10.1%), CINAHL (N = 24 entries, 8.08%), Psycinfo (N = 12 entries, 4.04%), Google Scholar (N = 7 entries, 2.35%), CENTRAL (N = 5 entries, 1.68%), Science Direct (N = 4 entries, 1.35%), Grey Literature (N = 2 entries, 0.67%), AMED (N = 2 entries, 0.67%), ERIC (N = 1 entries, 0.34%), Web of Knowledge (N = 1 entries, 0.34%), DARE (N = 1 entries, 0.34%), ProQuest (N = 1 entries, 0.34%), Cambridge Core (N = 1 entries, 0.34%), Oxford Academy (N = 1 entries, 0.34%), AgeLine (N = 1 entries, 0.34%), Scielo (N = 1 entries, 0.34%), and LILACS (N = 1 entries, 0.34%).

### Summary of evidence according to each cluster of health outcomes

#### Mediterranean diet and age-related chronic diseases

This health outcomes cluster included a number of physiologically age-related conditions and comorbidities of aging, that is, age-related macular degeneration (AMD) [22, 27, 60], bone demineralization (as measured by bone mineral density or BMD) [26], chronic obstructive pulmonary disease [26, 56], diabetic retinopathy [76], outcomes of physical dysfunction and decline (physical frailty [68, 71], loss of function[15, 50], muscle mass[50]and strength[50], functional disability [28], general physical health [28, 36], sarcopenia [28, 50], general quality of life dimensions (health-related quality of life or HRQL [65] and quality of life or QoL [67, 75]), osteoarthritis [72], rheumatoid arthritis [43, 69], sleep quality [28], and pain [28].

A total of 46 studies systematically analyzed the effect of MedDiet on AMD. Pameijer reported the use of antioxidant supplements and adherence to a Mediterranean diet, characterized by high consumption of vegetables, whole grains, and nuts and low consumption of red meat, were associated with reduced risk of early and late AMD progression [22]. Consistently, Gastaldello and colleagues reported greater adherence to a Mediterranean dietary pattern reduced the odds of developing AMD and the risk of progression to more advanced stages of the disease [27]. Chapman and colleagues, on the other hand, conducted a systematic analysis of 18 intervention studies comparing different dietary patterns (Mediterranean, Western, and Eastern) concluding that MedDiet reduced the risk of AMD progression, while an Eastern dietary pattern performed better than a Western dietary pattern with respect to association with AMD prevalence [60].

On the bone demineralization condition, a single report by Noori and colleagues [26] analyzed observational studies with a random-effects dose–response meta-analysis to estimate the change in total and region-specific BMD for a 2-point increase in MedDiet adherence score and to clarify the shape of these associations. Seven cross-sectional studies and one cohort study with a total number of 13,209 participants were included in the final analyses. A positive linear relationship was found between MedDiet adherence score and BMD of the hip and trochanter. A nonlinear relationship was observed for the lumbar spine, femoral neck, and whole body, with a sharper increase in BMD at the lowest MedDiet scores. Of note, associations remained significant after controlling for important confounding factors, including body weight, physical activity, smoking status, and energy intake. Thus, adopting a Mediterranean style eating pattern may have modest beneficial effects on bone health.

Data on COPD as an outcome came from a single report by Parvizian and colleagues [56] including 8 observational studies subjected to systematic review and random-effects meta-analysis. The authors here concluded that consumption of a healthy, Mediterranean eating pattern was associated with a lower risk of COPD (OR 0.88, 95% CI 0.82–0.94), while consumption of unhealthy eating patterns was associated with a higher risk of COPD (OR 1.22, 95% CI 0.84–1.76).

Data on diabetic retinopathy were analyzed across 31 studies (3 interventional, 28 observational) by Wong and colleagues [76]. The findings showed higher intakes of dietary fiber, fatty fish, and greater adherence to a Mediterranean diet to be protective outcomes against diabetic retinopathy. In contrast, high total caloric intake was associated with a higher risk of this outcome. The association between MedDiet adherence and physical decline has been thoroughly and systematically analyzed in 7 reports, constituting an important body of evidence. Kojima and colleagues [71] analyzed four longitudinal studies (5,789 older people with a mean follow-up of 3.9 years) that had physical frailty, a known multifactorial syndromic condition of aging, as a health outcome. Greater adherence to MedDiet (as measured by the MDS score, i.e., Mediterranean diet Score) was associated with a significantly lower risk of incident frailty (pooled OR = 0.62, 95% CI 0.47–0.82, P = 0.001 for MDS 4–5; pooled OR = 0.44, 95% CI 0.31–0.64, P < 0.001 for MDS 6–9) compared with poor adherence (MDS 0–3). Papadopoulou and colleagues [15] analyzed ten articles including four cross-sectional and six prospective studies. Here, adherence to the Mediterranean diet had in the over-65 s a positive role on muscle mass and function, while the results were less clear regarding muscle strength.

Coelho-Júnior and colleagues [36] from their analysis of nineteen cross-sectional studies that looked at 19,734 community-dwelling and institutionalized older adults without disabilities concluded high adherence to MedDiet cross-sectionally associated with physical performance. Granic and others [50], consistently, reported on the consumption of specific foods in the Mediterranean model, concluding vegetable consumption determined superior muscle strength and physical function. Further, combined vegetable and fruit consumption was significantly associated with a reduced risk of sarcopenia.

Moving toward measures of quality of life (QoL), three reports we found have systematically reviewed the literature on the effect of MedDiet with respect to this outcome. Wu and colleagues [65], in a dose–response relationship between diet exposure and health-related quality of life (HRQL), indicated that unhealthy eating behavior or lower diet quality was associated with decreased health-related quality of life among children and adolescents. Govindaraju and colleagues [67] pointed to healthy eating patterns as being associated with better self-assessment of health and QoL in one or more domains, and adherence to healthy eating patterns such as the Mediterranean diet was significantly associated with improvement in at least one of the domains of QoL. Padilha and colleagues [75], similarly, found MedDiet to have a correlation with quality of life, as well as with inflammatory status and cardiac function, but only in cross-sectional studies.

Regarding the effect of MedDiet on osteoarthritis, the prevalence of this condition, as analyzed by Morales-Ivorra and colleagues [72], was lower in participants with higher adherence to MedDiet. Biomarkers of inflammation and cartilage degradation related to osteoarthritis were also analyzed, and significant differences were found only for IL1-α, which was decreased in the MMedDietD group. In conclusion, three studies included in this systematic report demonstrated some relationship between osteoarthritis and MedDiet.

In the context of autoimmune inflammatory diseases, two systematic reports considered the effect of MedDiet on rheumatoid arthritis. Forsyth and colleagues [69] analyzed four studies including two intervention studies that reported an improvement in the visual analog scale of disease-associated pain and a decrease in the health assessment questionnaire score for rheumatoid arthritis in the Mediterranean diet groups. Only one study reported a 28 joint count reduction in disease activity score for rheumatoid arthritis for the Mediterranean diet group. This review identified the beneficial effects of the MedDiet in reducing pain and increasing physical function in people with rheumatoid arthritis. Schönenberger consistently reported that anti-inflammatory diets led to a significant reduction in pain compared to ordinary diets for inflammatory disease [43]. Last, a MedDiet has been shown to increase the quality of life, sleep, and well-being [28].

#### Mediterranean diet and cancer

The effect of MedDiet on cancer found eight reports of level I evidence so far conducted in the literature. On this topic, Verberne and colleagues [94] reviewed the evidence on the association between MedDiet patterns and cancer risk in observational epidemiological studies. Of the 12 studies included (7 cohort, 5 case–control), 10 studies (6 cohort, 4 case–control) provided some evidence that MedDiet was associated with a reduced risk of cancer incidence or mortality. The outcomes of the case–control studies were breast, endometrial, mouth/pharynx, esophageal, colorectal, and laryngeal cancer. The three Italian case–control studies [98] found strong significant inverse associations between MedDiet and the 3 upper aero-digestive tract cancers. A 1-point increase in MDS (Mediterranean diet score) was associated with a reduction in the risk of oropharyngeal, esophageal, and laryngeal cancer by 23%, 28%, and 29%, respectively. For colorectal cancer (CRC), a case–control study [99] found that following current U.S. dietary recommendations or a Mediterranean dietary pattern was associated with a 21% risk reduction in men. The two case–control studies on breast cancer found a reduction in breast cancer risk with increasing adherence to a dietary pattern [100, 101]. A 24% reduction in breast cancer incidence was observed among Hispanic and non-Hispanic American women in the highest quartile of MedDiet consumption analyzed by factor analysis. Similarly, a 35% lower risk of breast cancer was observed among Asian American women with the highest MedDiet scores (more than 8 points) compared with the lowest (0–3 scores). Authors also found a reduced risk of endometrial cancer for increasing adherence to the Mediterranean diet [102], with an over 50% risk reduction for women in the highest vs the lowest score.

The effect of MedDiet on CRC has shown a large body of evidence and consistency in the direction of the association, in a protective sense for those most adherent to MedDiet. Supporting studies include three systematic reviews. Potter and colleagues[86] found eight studies that examined the association between diet quality and CRC; except for one, they all reported a relationship between higher dietary scores and lower CRC risk. Steck and colleagues [87] identified five case–control and seven prospective cohort studies conducted in the United States and Europe. Five studies examined the MDS, four the HEI (Healthy eating Index) and four the DII (Dietary Inflammatory Index). Comparing the higher and lower score groups, higher MDS scores were associated with an 8–54% lower risk of CRC and higher HEI scores were associated with a 20–56% lower risk of CRC. Scores of a more proinflammatory diet were associated with a 12–65% higher risk of CRC than a more anti-inflammatory diet in studies that used DII. Results reported by sex suggested similar associations for men and women. Last, the report from Ubago-Guisaldo [35] concluded higher fish consumption and lower consumption of red and processed meat were related to a lower risk of CRC.

Other consistent evidence was found on breast and gastrointestinal cancer outcomes, out of which one report was on pancreatic cancer. Dianatinasab and colleagues [53] analyzed 10 studies on the association between diet and IDC (invasive ductal carcinoma) and ILC (invasive lobular carcinoma). This systematic review with meta-analysis provided evidence on the inverse association between MedDiet and risk of breast IDC and ILC and instead on the direct association between WD (Western diet) and increased risk of IDC and ILC.

Xiao and colleagues [66] examined associations between different dietary patterns and breast cancer risk by conducting a review with a meta-analysis of observational studies. The results suggested a possible increased risk of breast cancer associated with a Western dietary pattern and a reduced risk with a conservative, Mediterranean dietary pattern. Noteworthy, the inverse association between a prudent dietary pattern and breast cancer was significant in premenopausal but not postmenopausal women, and significant for both hormone receptor-positive and hormone receptor-negative cancers. Bloomfield and colleagues [81] reported on the large PREDIMED study, which found a lower risk of breast cancer in the two MedDiet groups combined compared with the control group (HR, 0.43 and CI, 0.21–0.88). Similarly, the systematic review by Potter and colleagues [86] inferred from the eight studies analyzed a relationship between diet quality and breast cancer risk in adult women, with higher MedDiet dietary scores associated with lower risk.

On gastrointestinal cancer outcomes, Moazzen [51] indicated higher quality diets significantly associated with a reduced risk of higher gastrointestinal cancers, yielding odds ratios of 0.59 (95% confidence interval: 0.48–0.72) for the Diet Inflammation Index, pooling results from nine studies, and 0.72 (95% confidence interval: 0.61–0.88) for the MedDiet score, pooling results from 11 studies.

In this context, we found two systematic reports on MedDiet and pancreatic cancer. Of these, Nucci[16] analyzed eight articles showing that higher adherence to MedDiet was associated with a lower risk of pancreatic cancer (HR:0.82 and CI 0.76–0.88, p < 0.001) based on 1,301,320 subjects. Accordingly, Gianfredi and colleagues [29] analyzed 23 articles on the same topic finding convincing or suggestive evidence for a healthy/prudent, plant-based, fruit- and vegetable-based diet and a lower risk of pancreatic cancer, while a high intake of red meat was associated with a higher risk of pancreatic cancer at a convincing level of evidence.

#### Mediterranean Diet and CVD

In the present study, the association between MedDiet and CVD outcomes was analyzed for measures found through the selected studies and including blood pressure [38, 49, 52] (systolic [24, 54, 55, 77, 85], diastolic [24, 39, 54, 55, 77, 85]), the resistance of the uterine and umbilical arteries [63], myocardial infarction [81], CHD [93, 96], cardiac function as estimated by left ventricular ejection fraction or left atrial ejection fraction [75], and endothelial function as estimated by flow-mediated dilation (FMD) [14, 46, 49, 90]. Of these, blood pressure and FMD gathered a larger body of evidence.

As for blood pressure, Papadaki and colleagues [49] examined a cluster of studies including a total of 36,983 subjects. Here, MD resulted in greater beneficial changes in 18 of 28 components and risk factors of metabolic syndrome, including systolic and diastolic blood pressure, and lower risk of cardiovascular disease incidence. Abbate [52] concluded that a low-fat intervention diet appeared effective only when combined with moderate-intensity [49] exercise and weight loss, while a Mediterranean diet intervention without physical activity reduced both systolic and diastolic blood pressure, the rate of major CV events, and the risk of developing type 2 diabetes. Garcia [85] conducted a systematic review with a meta-analysis of 29 intervention studies finding that traditional MedDiet had significant beneficial effects for five of six metabolic risk factors, including systolic and diastolic blood pressure. The same for Malakou [77], with 4 of 7 reports analyzed favoring a significant protective effect of MedDiet on systolic and diastolic blood pressure. Rees [54] found moderate-quality evidence for a reduction in both systolic and diastolic blood pressure with a Mediterranean-type diet, while Luong [24] argued a Mediterranean dietary pattern resulted in a reduction in triglyceride levels and systolic blood pressure, while having no effect on diastolic blood pressure and glucose in the short term; however, other dietary patterns had inconclusive effects.

Prominent evidence was found for the effect of MedDiet on endothelial function Among them, Fatima [14] observed an inverse relationship between endothelial function and MedDiet intake. Overall, MedDiet increased FMD by 1.39% with significant improvement in endothelial function in both healthy patients and those with increased CVD risk. In line with the latter, Shannon [46] conducted a systematic review with meta-analysis including 14 articles reporting data on 1930 participants, and varied study duration from 4 weeks to 2 years. A beneficial effect of the MedDiet diet on endothelial function was observed with MedDiet interventions that had improved FMD by 1.66%, used as a reference method for noninvasive clinical measurement of endothelial function. Of note, the effects of the MedDiet diet on endothelial function were not changed by health status, type of intervention, study duration, study design, BMI, or age of participants. Also interesting, an analysis of 86 studies conducted by Reijnders [63] showed adequate nutrition in the first trimester, periconceptional folic acid supplementation and strong adherence to an MD were associated with lower uterine and umbilical artery resistance in the second and third trimesters.

Some protective roles of MedDiet on the risk of CHD and myocardial infarction were found through three systematic reports. From the analysis of three secondary prevention studies reporting cardiovascular outcomes, Bloomfield [81] found one attesting to a lower risk of recurrent myocardial infarction and cardiovascular death with MedDiet. Sofi [93] analyzed three studies that had the incidence and/or mortality from cardiovascular disease reduced by MedDiet. Mente [96] found strong supporting evidence for valid associations between protective factors, including intake of vegetables, nuts, and Mediterranean and high-quality dietary patterns with CHD, and associations between harmful factors, including intake of trans fatty acids and foods with high glycemic index or load.

#### Mediterranean Diet and diabetes

Adhering to MedDiet Also means promoting better-controlled blood glucose balance in terms of fasting blood glucose, HbA1c, insulin resistance, and risk of developing diabetes, as we found from the cluster of systematic reviews found about this health outcome.

Among the above parameters, substantial evidence emerged from up to eight systematic review papers that found benefits in fasting blood glucose for individuals most adherent to the Mediterranean model. Sepandi and colleagues [25] included in their report a number of papers evaluating diet with a *priori* indices; most of the papers that considered the Mediterranean diet Score (MDS) index in this report suggested that increasing the MDS score would lead to a significant reduction in HbA1C, fasting blood glucose, and 2 h postprandial blood glucose. Papadaki and colleagues [49] investigated the effect of MD on metabolic health by analyzing RCTs in adults. The effect of MD on blood glucosee was greater even when the studies did not supplement foods, were conducted in Mediterranean versus non-Mediterranean countries, and when the duration of the intervention was ≥ 6 months, compared with a shorter time. Malakou and colleagues [77] investigated the combined effect of MD promotion and physical activity on metabolic risk factors in adults by taking RCTs. Changes between groups were reported from pre- to post-dietary intervention for fasting blood glucose in seven studies. Effect estimates suggested that the combined effect of MedDiet and physical activity had resulted in greater reductions in HOMA-IR index and fasting blood glucose. Grosso and colleagues [77] reviewed the most recent evidence on the health value of MedDiet against CVD and its relationship to cardiovascular risk factors. Results from the dynamic SUN cohort, the EPIC study, the ATTICA study, as well as several nutrition surveys such as ENCA, NHANES III, and single-center studies, which collectively examined more than 1,000,000 subjects residing in Mediterranean and non-Mediterranean countries, were considered. The data reviewed suggested that the health benefits of the MedDiet model are mainly due to the existence of biological interactions among its different components rather than the effect of a single food or nutrient group. Regarding CVD, highlighted mechanisms underlying the protective effects include improvements in lipid and glucose profiles, and insulin resistance. In the meta-analysis by Garcia and colleagues [85], the goal was to obtain effect sizes for metabolic risk factors and to explain variability in the current literature based on study design, sample, and dietary characteristics. Significant effects in favor of MedDiet were found for a number of metabolic markers, including fasting blood glucose. MedDiet was found significantly beneficial when the intervention was of longer duration, was conducted in Europe, used a behavioral technique, and was conducted in small groups. Brown [95] conducted a systematic review of controlled studies of lifestyle interventions in adults with a body mass index less than 35 kg/m^2^ with at least 2 years of follow-up.

There was an associated improvement in triglycerides and fasting plasma glucose in two studies but not in cholesterol or HbA1c. Abbate [52] and Silveira [38] confirmed the same beneficial trend for MedDiet on CVD risk factors including altered glucose metabolism parameters.

Four systematic review reports were found to have outcome incidence of type 2 diabetes mellitus. Data from some RCTs were analyzed by Bloomfield [81] suggesting that unrestricted MedDiet on fat intake may be associated with reduced incidence of cardiovascular events and type 2 diabetes mellitus compared with any other diet but does not affect all-cause mortality. Koloverou and colleagues [81] reported the effect of the Mediterranean diet on the development of type 2 diabetes mellitus analyzed by meta-analysis of 10 prospective studies and 136,846 participants. Results indicated greater adherence to the Mediterranean diet was associated with a 23% reduced risk of developing type 2 diabetes (combined relative risk for the upper versus lower centile). Subgroup analyses based on region, participant health status, and a number of confounding factors controlled for showed similar results. Also on MedDiet and risk of type 2 diabetes, a systematic review and dose–response meta-analysis of prospective cohort studies was also retrieved [31, 81, 89, 90]. Fourteen prospective cohort studies (410,303 participants and 41,466 cases) were included, and an inverse association was found between the highest and lowest categories of MedDiet adherence and for a 2-point increase in MedDiet diet adherence score. A single report [78] analyzed gestational diabetes as an outcome in relation to MedDiet or other diets. From this report, diets such as MedDiet, the DASH (Dietary Approaches to Stop Hypertension) diet, and the AHEI (Alternate Healthy Eating Index) diet were associated with a 15–38% reduced relative risk of gestational diabetes. In contrast, frequent consumption of potatoes, processed meat/meats, and protein (% energy) derived from animal sources was associated with an increased risk of gestational diabetes.

### Mediterranean Diet and inflammation

Regarding the effect of MedDiet on inflammatory status, we collected one report on Platelet-Activating Factor (PAF) [73], three on TNF-alpha [19, 74, 75], seven reports on interleukin-6 (IL-6) [19, 41, 49, 57, 74, 75, 90], and seven on C-reactive protein (CRP) [38, 41, 49, 52, 74, 84, 90], showing all beneficial results of Mediterranean diet pattern.

Mayr [74] analyzed observational studies finding significant inverse associations between Mediterranean-type diet scores and inflammatory cytokines. Five clinical studies (4 in non-Mediterranean countries) showed nonsignificant reductions, while 2 studies conducted in Spain showed significant reductions in C-reactive protein with a Mediterranean-type diet. MedDiet also showed a correlation with inflammation in Dos Reis Padilha’s report [75], but only in cross-sectional studies. Instead, decreases in BMI, TNF-α, IL-6, and CRP, most of them significant, were reported in the reports by Moore [19] on the population with overweight or obesity. Analysis of randomized, prospective studies by Genel [57] showed that a low-inflammatory diet such as MedDiet is associated with greater weight loss, greater decreases in inflammatory biomarkers, greater improvement in measures of pain, and greater improvement in measures of physical function than the usual diet. Instead, Hart and colleagues [41] collected reports in which the most frequently assessed biomarkers were CRP (64 reports) and/or IL-6 (22 reports). At the cross-sectional level, most analyses reported an association between higher dietary scores (mostly Mediterranean and anti-inflammatory diet scores) and lower inflammatory markers, with 82 significant associations from 133 analyses. Consistent with precedent, consumption of a healthy dietary pattern was associated with a significant reduction in CRP in an additional report from Neale [84]. Non-significant changes were found for all other biomarkers. This research group found evidence for the favorable effects of healthy food patterns on CRP, with limited evidence for other biomarkers.

#### Mediterranean Diet and kidney health

Regarding renal health, our research found evidence of renal function markers such as proteinuria and eGFR. Quintela and colleagues [37] evaluated the association of dietary patterns with the development and progression of chronic kidney disease (CKD) in observational studies. A significant association was observed between unhealthy eating patterns and an increased risk of developing or worsening CKD, as well as an adverse effect. While healthy dietary patterns, characterized by the consumption of fruits, vegetables, and dietary fiber, showed nephroprotective results. An additional analysis of prospective studies took renal outcome assessed as eGFR [64]. A total of twenty-six research articles were included and concluded that adherence to DASH and MedDiet diets was significantly associated with reduced risk of CKD in most studies. In addition, retrospective “unhealthy” dietary patterns were associated with an increased risk of CKD.

#### Mediterranean Diet and liver health

Two reports evaluated liver fat content as a means of assessing liver health in relation to exposure to the Mediterranean model. Reduction in hepatic steatosis was statistically significant in 3/5 MedDiet interventions assessed by Saeed and colleagues [62]. Most of the data appear to support MedDiet-based interventions, although further randomized trials are needed to evaluate comparative efficacy for NAFLD. Angelidi and colleagues [23] found moderate evidence that a low-carbohydrate diet, compared with a low-calorie diet, and the Mediterranean diet, compared with a low-fat, high-carbohydrate diet resulted in greater reductions in hepatic fat content. A post hoc analysis, which included two eligible studies that evaluated the effect of the Mediterranean diet compared with a low-fat diet, regardless of the presence of diabetes at baseline, showed strong evidence that the Mediterranean diet reduces hepatic fat content and triglyceride concentrations.

A total of four systematic review reports were found instead to analyze MedDiet in relation to serum transaminase levels. Among these, Sangouni [21] and colleagues systematically reviewed and meta-analyzed RCTs investigating the effect of MedDiet on liver enzymes. Ten RCTs (n 705 participants) that evaluated the impact of MedDiet on liver enzymes, including aspartate aminotransferase (AST), alanine transaminase (ALT) and γ-glutamyltransferase (GGT), were included. The results showed that the MedDiet diet significantly reduced AST and GGT but had no significant effect on ALT.

#### Mediterranean Diet and mortality

Of the selected systematic review reports, one study assessed breast cancer mortality [30], two studies assessed cardiovascular mortality [54, 81], one study assessed cancer-related mortality [48], and two studies assessed the overall mortality [30, 34, 54] as an outcome related to MedDiet exposure. Healthy eating and mortality among breast cancer survivors was the focus of the systematic review by Lee and colleagues [30]. The analysis included 11 publications from eight cohorts with data from 27,346 survivors and seven dietary indices. Improved diet quality after diagnosis significantly reduced all-cause mortality by 21% and marginally reduced breast cancer-specific mortality by 15%. Three studies included the MDS as a priori index, and the meta-analysis showed a significantly reduced risk for all-cause mortality and a reduced but not significant risk for breast cancer-specific mortality. Rees and colleagues[54] analyzed the Mediterranean dietary intervention versus another dietary intervention for primary prevention. This research group targeted the PREDIMED study being the only one to report clinical events for this comparison. This study was retracted and reanalyzed because of doubts about randomization at two of the 11 sites and the inclusion of non-randomized second family members. The evidence showed little effect of the intervention (advice to follow a Mediterranean diet plus extra virgin olive oil or nut supplementation) versus a low-fat diet on CVD mortality or all-cause mortality (HR 1.0, 95% CI 0.81 to 1.24) over 4.8 years. The same report also included studies of Mediterranean dietary intervention versus usual care for secondary prevention. Targeted here was the Lyon Diet Heart Study that had examined the effect of advice to follow a Mediterranean diet and supplement canola margarine versus usual care in 605 CHD patients over a 46-month period; low-quality evidence emerged of a reduction in adjusted estimates for CVD mortality (HR 0.35, 95% CI 0.15–0.82) and all-cause mortality (HR 0.44, 95% CI 0.21–0.92) with the intervention. According to Bloomfield [81], of three secondary prevention studies that reported cardiovascular outcomes, however, only one found a lower risk of recurrent myocardial infarction and cardiovascular death with the Mediterranean diet.

Pooled estimates of associations between different dietary interventions and various cancer outcomes were obtained by applying a random-effects meta-analysis in the report from [48]. The few available studies on the plant-based, Mediterranean diet failed to support its potential to prevent overall cancer mortality compared with a nonvegetarian diet (e.g., pooled hazard ratio (HR) = 0.97; 95% confidence interval (CI) 0.88–1.06), however, the association between adherence to the Mediterranean diet and cancer mortality reached statistical significance (e.g., pooled HR = 0.84; 95% CI 0.79–0.89). Tang and colleagues [34] evaluated the effect of MedDiet on mortality in subjects at risk of CVD. Seven cohort studies (eight data sets) with a total of 37,879 participants with a history of CVD were eligible for the main analysis. Pooled hazard ratios were 0.85 (95% CI 0.78–0.93; n = 8) for all-cause mortality and 0.91 (95% CI 0.82–1.01; n = 4) for cardiovascular mortality for each 2-unit increase in MedDiet adherence score. Subgroup analyses for all-cause mortality showed that the association appeared relatively stronger in Mediterranean areas (HR = 0.76 [0.69–0.83]) than in non-Mediterranean areas (HR = 0.95 [0.93–0.98]) and in studies with shorter duration (HR = 0.75 [0.66–0.84] for < 7 years vs. HR = 0.94 [0.91–0.98] for ≥ 7 years). No evidence of publication bias was observed. In conclusion, this report of prospective cohort studies provided evidence that adherence to MedDiet improves survival in people with a history of CVD.

#### Mediterranean Diet and neurological diseases

A series of neurological outcomes have been clustered according to retrieved papers in our analysis. These included brain-derived neurotrophic factor (BDNF) as a marker able to stimulate the survival and differentiation of certain neurons and synapses belonging to the central nervous system and peripheral nervous system [70], global cognitive function, cognitive decline, depression, dementia, Alzheimer’s disease, Parkinson’s disease, and a number of neurological domains (verbal and visual memory, processing speed, working memory, language, and executive function.

Cognitive decline was analyzed in the report by Townsend [18] where most prospective studies (77%) examined a priori dietary patterns, with the Mediterranean diet examined most frequently. 52% of prospective studies and 50% of RCT studies reported a protective relationship between “healthy” dietary patterns and overall cognitive decline. Overall, 59% of prospective studies reported positive associations between healthy dietary patterns and the risk of cognitive impairment. Instead, Bianchi [42] found that MedDiet, nutritional support, and calorie-controlled diets play a protective effect against cognitive decline, Alzheimer’s disease, and Parkinson’s disease, while malnutrition and insulin resistance are significant risk factors. Malnutrition also activates dysfunction of the gut-microbiota-brain axis, which exacerbates the neurogenerative process. Again, the main outcomes of Limongi and colleagues’ review [47] were cognitive decline, cognitive performance, and function, Mild Cognitive Impairment (MCI), Alzheimer’s disease (AD), and dementia. Forty-five of the 995 retrieved articles were analyzed of which seven were RCTs and 38 were longitudinal studies. Overall, the studies showed that MedDiet has some protective effects on cognitive decline. Regarding cognitive domains, MedDiet diet was only associated with improved global cognition. Results have been mixed for MCI and AD.

As for dementia, Aridi [79] was found to collect evidence-based data examining the effect of MedDiet adherence on cognitive function and the risk of developing dementia or Alzheimer’s disease. Cross-sectional and cohort studies in non-Mediterranean regions showed mixed results. However, cohort studies in the Mediterranean region and randomized controlled trials showed more cohesive results of the beneficial effect of MedDiet on cognitive function. The authors concluded that MedDiet might play an important role in cognitive health and risk of Alzheimer’s disease and dementia. Altun and colleagues [58] qualitatively reviewed the results of twenty observational studies and six intervention studies. Most (85%) of the observational studies supported evidence that the Mediterranean dietary pattern is associated with reduced depressive incidence, and all intervention studies echoed these findings. Several measures of adherence to the Mediterranean diet, the Healthy Eating Index (HEI) and Alternative HEI (AHEI), the Dietary Approaches to Stop Hypertension, and the Dietary Inflammatory Index were analyzed in the report by Lassale and colleagues [59]. The most convincing evidence was found for the Mediterranean diet and incident depression, with a combined relative risk estimate of the highest versus lowest adherence category from four longitudinal studies of 0.67 (95% CI 0.55–0.82). A lower dietary inflammatory index was also associated with a lower incidence of depression in four longitudinal studies (relative risk 0.76; 95% CI 0.63–0.92).

Meta-analysis of studies selected by Sofi and colleagues [93] with a random-effects model showed that a 2-point increase in MedDiet adherence was associated with a significant reduction in overall mortality and neurodegenerative diseases (RR = 0.87; 95% CI 0.81, 0.94). Longitudinal results from Coelho-Júnior and colleagues [36] on thirty-four prospective studies with a mean follow-up period ranging from 3.0 to 12.6 years and examining 98,315 community residents indicated high MedDiet scores induced less decline in global cognition. Along these lines, McBean [32] concluded that an MedDiet dietary intervention had a significant effect on memory and executive function. When analyzing stroke as an outcome, Liyanage [82] found combined a number of studies that gave evidence of MedDiet for protection against major vascular events (RR 0.63, 95% confidence interval 0.53–0. 75), coronary events (0.65, 0.50–0.85), stroke (0.65, 0.48–0.88) and heart failure (0.30, 0.17–0.56) but not for all-cause mortality (1.00, 0.86–1.15) or cardiovascular mortality (0.90, 0.72–1.11). The association between dietary patterns and stroke was also reviewed in a study based on a total sample of 195,875 participants. The data showed a consistent protective effect of increased adherence to the Mediterranean diet on stroke incidence (pooled relative risk 0.68, 95% CI 0.58, 0.79). Thus, a healthy dietary pattern benefits stroke incidence and mortality, adding a new direction toward stroke prevention at the population level.

Of note, we found a report from Gregory [17] regarding seven articles on 21,933 participants. Four studies reported on hippocampal volume, with inconclusive or no associations with MedDiet adherence. Two studies found a significant association between higher MedDiet adherence and lower WMHV, while two other studies found no significant associations.

#### Mediterranean Diet, obesity, and related metabolic features

About the cluster of health outcomes related to obesity and related dysmetabolic conditions, including metabolic syndrome and its individual construct domains, the present umbrella systematic review found a range of evidence in favor of MedDiet as a strategic nutritional intervention in terms of protection and risk reduction.

Several level-I evidence papers in favor of MedDiet were found for measures of waist-to-hip ratio, BMI, body fat, and waist circumference, well-known as clinical parameters of wide use as proxies for overweight phenotypes and general nutrition status.

Moore [103] found that consuming a MedDiet implied reductions in BMI, TNF-α, IL-6 and serum CRP among the most significant results. Thackrey [20] observed significant weight loss within the groups for a low-fat diet, Mediterranean diet, fasting, LCD with fasting, intermittent fasting, or continuous energy restriction. Malakou [77] found that, compared to a control diet, there was strong evidence of a beneficial effect of MedDiet promotion and physical activity on body weight (− 3.68 kg, 95% CI − 5.48 to − 1.89) on body mass index (− 0.64 kg/m2, 95% CI − 1.10, − 0.18), waist circumference (− 1.62 cm, 95% CI − 2.58, − 0.66), total cholesterol (− 6.30 mg/dL, 95% CI − 9.59, − 3.02) and HDL cholesterol (+ 3.99 mg/dL, 95% CI 1.22, 6.77). Same lines, higher diet quality was associated with relatively lower prospective weight gain as well as a lower risk of becoming overweight or obese, compared with poor diet quality in the report by Aljadani [88]. In the earliest available report retrieved [97], however, the results indicate a possible role of MedDiet in the prevention of overweight/obesity, and physiological mechanisms may explain this protective effect. Bakaloudi and colleagues [33] presented a review of 58 studies, showing that waist circumference and triglycerides were significantly lower in the high adherence MedDiet group (SMD: − 0.20, (95%CI: − 0.40, − 0.01), SMD: − 0.27 (95%CI: − 0.27, − 0.11), respectively), while HDL cholesterol was significantly higher in the same group (SMD: − 0.28 (95%CI: 0.07, 0.50). MedDiet had an overall positive impact on all metabolic syndrome parameters.

The same conclusions were drawn from data presented by Garcia [85] on parameters of waist circumference, triglycerides, blood glucose, systolic blood pressure and diastolic blood pressure. Here the MedDiet was significantly beneficial when the intervention was of longer duration and was conducted in Europe. The results from Grosso and colleagues[90] indicated that adherence to a modified MedDiet, rich in vegetables and unsaturated fatty acids, is associated with lower abdominal adiposity as measured by waist circumference without being significantly associated with BMI. Most of the interventions analyzed by Abbate [52] had found to improve at least some markers of CV risk, and the greatest improvement was achieved with increased time spent on physical activity. A low-fat intervention diet appeared effective only when combined with moderate-intensity exercise and weight loss, while a Mediterranean diet intervention without physical activity reduced systolic and diastolic blood pressure, the rate of major CV events, and the risk of developing type 2 diabetes. The effect of the Mediterranean diet compared with a low-fat diet, regardless of the presence of diabetes at baseline, showed strong evidence in the report by Angelidi [23] that the Mediterranean diet reduced liver fat content (− 4.1%, 95% CI − 5.8 to − 2.3, P < 0.001; I^2^ = 0%) and triglyceride concentrations (− 16.9 mg/dL, 95% CI − 26.3 to − 7.7, P < 0.001).

### Evaluation of the quality of evidence

**Fig. 3:**
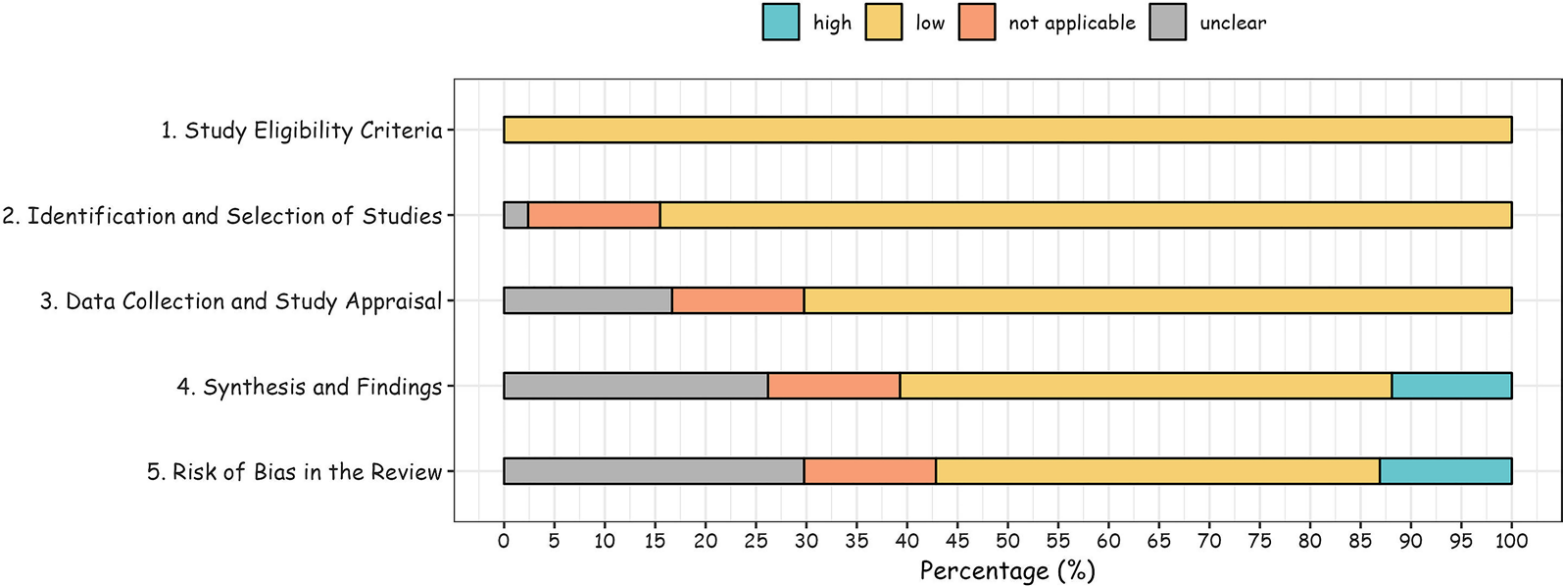
Bar chart showing the survey developed by the ROBIS tool to examine publication bias among the different studies included

A bar plot was built as a visual tool to show the survey derived from the ROBIS tool to examine publication bias across the different studies taken on board (Fig. 3). The results showed the following risk of bias distribution across the 5 questions: low risk (100%, N = 84 entries) or not applicable (13.09%, N = 11 entries) for question 1 related to study eligibility criteria; low risk (84.5%, N = 71 entries) or not applicable (13.09%, N = 11 entries) for question 2 related to identification and selection of studies; low risk (70.2%, N = 59 entries), unclear (16. 6%, N = 14 entries), or not applicable (13.09%, N = 11 entries) for question 3 related to data collection and study appraisal; high risk (11.9%, N = 10 entries), low risk (48.8%, N = 41 entries), not applicable (13.09%, N = 11 entries), or unclear (26. 19%, N = 22 entries) for question 4 on synthesis and findings; high risk (13.09%, N = 11 entries), low risk (44.04%, N = 37 entries), not applicable (13.09%, N = 11 entries), or unclear (29.7%, N = 25 entries) for question 5 on risk of bias in the review. Drawing the sums in terms of the quality of this umbrella systematic review, the risk of bias across the analyzed studies was mostly low according to the five ROBIS domains, i.e., low risk for 86.9%, 84.5%, 70.2%, 48.8%, 44.04% of the studies ranging from domain 1 to domain 5 of the questions, respectively. Of note, only a very small percentage (N = 11, allocated as “not applicable” to ROBIS analysis) of studies could not be quality screened due to the authors’ lack of use of a validated and scientifically recognized tool for risk of bias assessment of the studies included in the review.

## Discussion

The present systematic umbrella review was undertaken to evaluate the scientific evidence on the wide range of human health benefits of adhering to the MedDiet model principles to support the scientific reliability of the one-health Med-Index FOP food labeling tool. A total of 84 studies of evidence level I were analyzed and clusterized by health outcomes into macro-categories (age-related chronic diseases, neurological diseases, obesity, and related metabolic characteristics (including metabolic syndrome), CVD, cancer, diabetes, systemic inflammation status, liver health, renal health, and mortality). The MedDiet was found to strongly benefit age-related chronic diseases (21.5%), neurological disorders (19%), and obesity-related metabolic features (12.65), followed by CVDs (11.4%), cancer (10.1%), diabetes (7.5%), liver health (6.3%), inflammation (5%), mortality (5%), and renal health (1.2%) [14–97]. The quality of the studies was moderate to high, according to ROBIS risk of the bias assessment tool.

Overall, the evidence from observational and intervention studies has consistently reported that the MedDiet diet is useful for the prevention of several chronic diseases, including CVD outcomes, cancer, and neurodegenerative diseases, among others. Deepening the foods of the Mediterranean model, greater emphasis was found on fruits and vegetablesas having a stronger impact on cognitive function than other food groups [104], while nuts on cardiovascular risk factors and CVD incidence and mortality. Also, olive oil as a mainstay of the MedDiet model has been strongly emphasized for several metabolic benefits, especially when substituted for other fats such as margarine or butter [105]. Not surprisingly, olive oil is a proven health superfood; over the past fifty years, a wealth of scientific evidence has accumulated that points to a plethora of direct and indirect benefits of olive oil and other derivative products, such as olives and olive leaves. Evidence regarding the health benefits of olive oil has been demonstrated for cardiovascular and metabolic systems, cancer prevention, high blood pressure, cholesterol levels, cognitive/neurological conditions, diabetes, inflammatory processes and oxidative stress, coagulation, and so on [106].

In light of these findings, and given the perspective of globalization and industrialization, as well as the rapid rise of fast-food chains and advances in the food industry that have turned the traditional MedDiet pattern into a more global and Westernized eating way, the adoption of a supplementary FOP food labeling system that could be easily and intuitively enjoyed by the consumer would greatly assist in making informed choices and curb this trend [107]. In fact, the intake of fresh fruits and vegetables, nuts, legumes, whole grains, and fish is increasingly going to be replaced by energy-dense processed foods rich in refined carbohydrates, sugar, and sugar-sweetened beverages, and animal or partially hydrogenated fats. These changes in diet, combined with a more sedentary lifestyle, both at work and at leisure, are primarily responsible for the increase in obesity and the CVD epidemic of recent decades. In this context, scientific papers supporting the benefits of MedDiet, and its components are essential to guide food policies to counter the burden of health diseases and individual decisions toward a more plant-based diet that is beneficial to human populations and sustainable for our planet as we move toward sustainability [108].

In light of these findings, the Med-Index FOP food label [7], which simultaneously integrates the nutritional and sustainability characteristics of foods [10], may work well as an objective reference for the use of the Mediterranean label on food products, as well as being a suitable tool for achieving the goals of the 2030 EU Agenda for sustainable development. Further, the UNESCO Working Group on Education for Health and Sustainable Development [109] is actually planning to raise a global possibility of promoting a healthy and sustainable food model, based on the nutritional properties of MedDiet but implemented at the local level using the food available in different areas of the world. In this context, “Planeterranean” is the term developed by the UNESCO Chair on Health Education and Sustainable Development to refer to this new sustainable dietary model, based on the nutritional properties of MedDiet, but implemented at by using the food products locally available in any part of the world, consistently with the Sustainable Development Goals (SDGs) set by the United Nations in the 2030 Agenda and the principles of the circular economy. Thus, a deep understanding of the MedDiet model supported by the Med-Index in food choices will be able to promptly focus the community's interest in proper food regimen, bring citizens closer to the productive world, stimulate the interest and curiosity of adults and youth in becoming aware of the importance of sustainability as a primary tool for sustainable development, laying the foundation for the creation of a network of sustainable and resilient communities.

## Conclusions

This systematic review demonstrates that human health can benefit from adhering to the MedDiet principles in multiple health domains. These principles can be addressed in any European region and worldwide by implementing MedDiet principles with locally available foods. Against this evidence, the Med-Index, as a one-health FOP food label proposed to meet the EU’s call for a tool that combines the nutritional, environmental and social dimensions of sustainability, could work well in bringing consumers closer to the healthy and sustainable Mediterranean model. However, experimental studies to corroborate the efficacy of using this food FOP are still awaited.

### Supplementary Information

**Additional file 1:** **Table S1.** Reported risk of bias (ROB) across selected studies.

## Data Availability

All data supporting the findings of this study are available from the corresponding authors upon reasonable request.

## Abbreviations

MedDiet: Mediterranean diet
FOP: Front-of-pack
PRISMA: Preferred Reporting Items for Systematic Reviews and Meta-analyses
RCT: Randomized controlled trial
BDNF: Brain-derived neurotrophic factor
AD: Alzheimer’s disease
PD: Parkinson’s disease
wc: Waist Circumference
HDL-c: HDL-cholesterol
LDL-c: LDL-cholesterol
VAT: Visceral adipose tissue
TC: Total cholesterol
WHR: Waist-to-hip ratio
CVD: Cardiovascular Disease
DBP: Blood pressure
SBP: Systolic blood pressure
FMD: Flow-mediated dilation
CHD: Coronary heart disease
CRC: Colorectal cancer
T2DM: Type 2 diabetes mellitus
FBG: Fasting blood glucose
PAF: Platelet-Activating Factor
IL-6: Interleukin-6
CRP: C-reactive protein
AST: Aspartate aminotransferase
GGT: Gamma-glutamyl transferase
ALT: Alanine transaminase
eGFR: Estimated glomerular filtration rate
BMD: Bone mineral density
COPD: Chronic obstructive pulmonary disease
HRQL: Health-related quality of life
QoL: Quality of life
HEI: Healthy eating Index
DII: Dietary Inflammatory Index
IDC: Invasive ductal carcinoma
ILC: Invasive lobular carcinoma
